# Supplementary material for: Nucleus Isthmi Is Required to Sustain Target Pursuit during Visually Guided Prey-Catching
Source: Curr Biol. 2019 Jun 3;29(11):1771–1786.e5. doi: 10.1016/j.cub.2019.04.064 (PMC6557330; doi:10.1016/j.cub.2019.04.064)
Supplement: Document S1. Figures S1–S6 [file mmc1.pdf]

Current Biology, Volume *29*

Supplemental Information

Nucleus Isthmi Is Required to Sustain Target Pursuit during Visually Guided Prey-Catching

Pedro M. Henriques, Niloy Rahman, Samuel E. Jackson, and Isaac H. Bianco

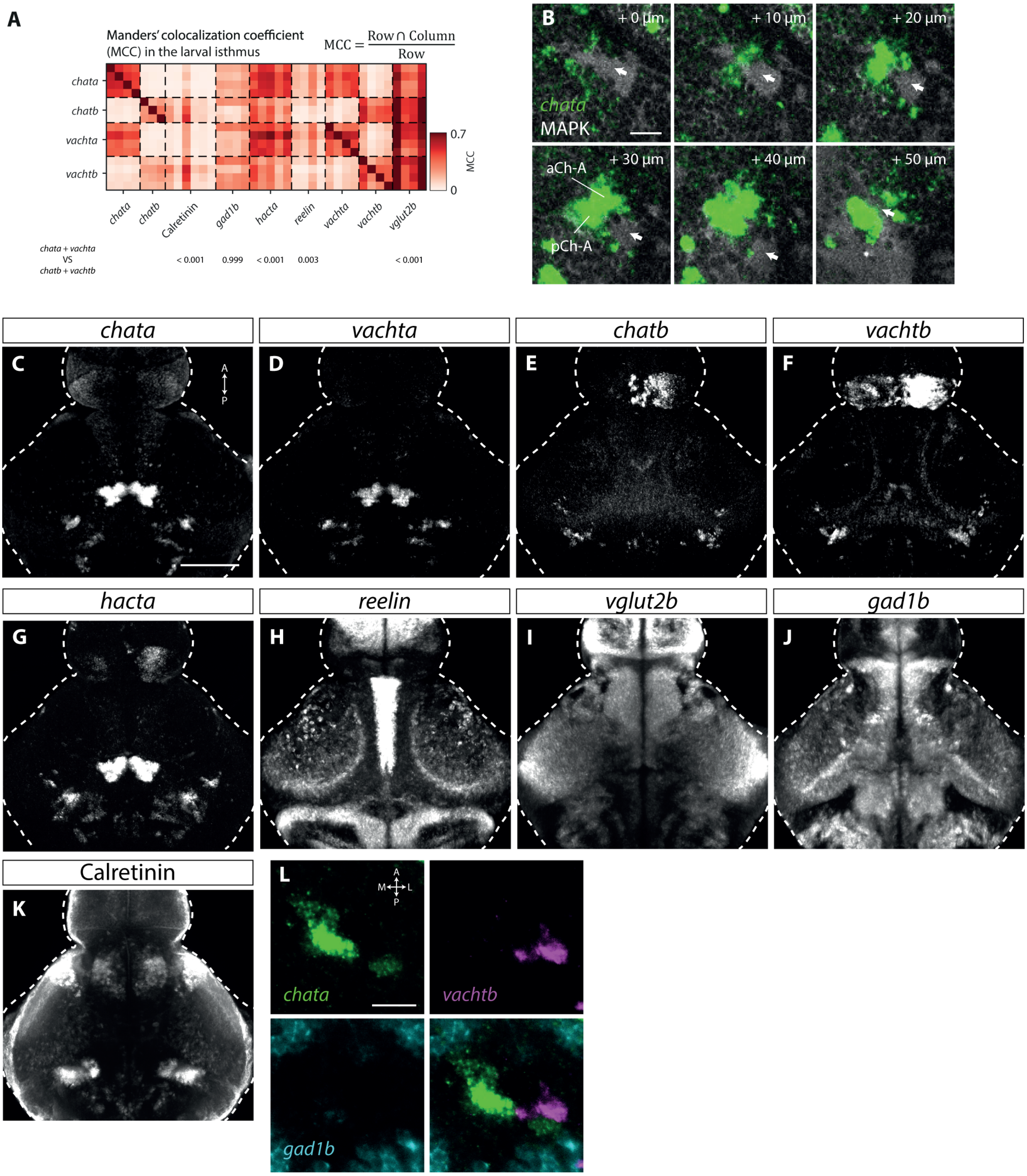

Figure S1: Isthmic gene expression, related to Figure 1. (A) Manders' colocalization coefficient (MCC) quantifying overlap of markers in the larval isthmus. This data shows all pairwise comparisons between single brains with mean values shown in Figure 1H. p-values correspond to comparisons of MCC for *chata+vachta* versus *chatb+vachtb* and the corresponding upper labels (Tukey's HSD correction for multiple comparisons following 2-way ANOVA). (B) MAPK expression in the isthmus showing the large cerebellar fascicle (white arrows) adjacent to and crossing *chata* expression. Scale bar, 25 μm. (C-K) Registered expression of markers used to characterize the larval isthmus. (C-H) show maximum-intensity projections across the entire dorso-ventral extent of the brain (325 μm), while (I-K) show projections across a smaller dorso-ventral region at the level of the isthmus (90 μm). All images show median intensity across registered datasets. Scale bar 100 μm. (L) Single focal plane from a triple fluorescent *in situ* of a 6 dpf larval brain detecting *chata*, *vachtb* and *gad1b* mRNA in the right isthmus. Almost no overlap is observed between these markers. Scale bar, 20 μm.

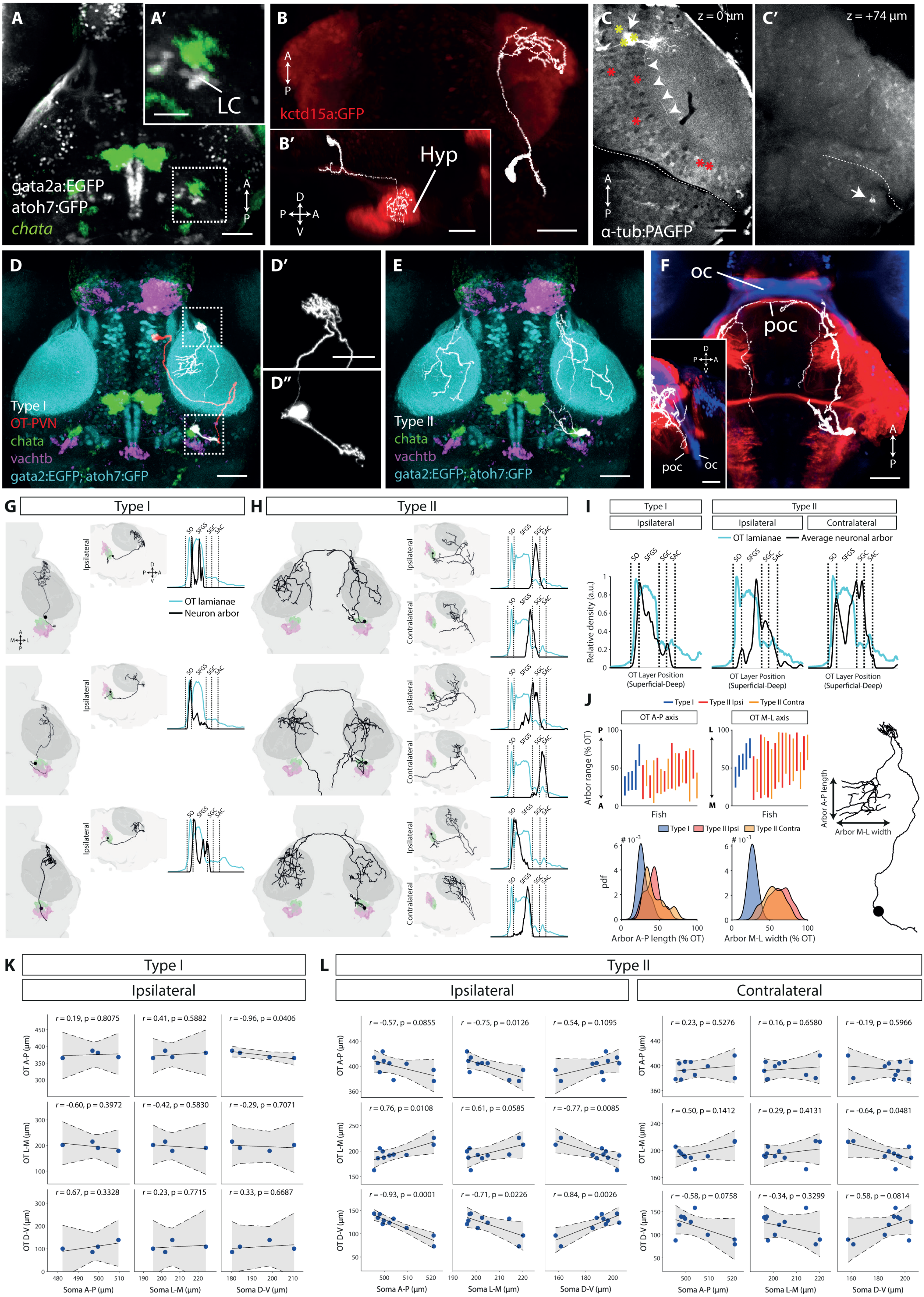

A
A′
LC
gata2a:EGFP
atoh7:GFP
chata
A
P
B
kctd15a:GFP
B′
Hyp
D
V
P
A
C
z = 0 μm
α-tub:PAGFP
C′
z = +74 μm
D
D′
D″
Type I
OT-PVN
chata
vachtb
gata2:EGFP; atoh7:GFP
E
Type II
chata
vachtb
gata2:EGFP; atoh7:GFP
F
OC
poc
poc
oc
G
Type I
Ipsilateral
SO
SFGS
SGC
SAC
OT lamianae
Neuron arbor
M
L
H
Type II
Ipsilateral
Contralateral
I
Type I
Type II
Ipsilateral
Ipsilateral
Contralateral
OT lamianae
Average neuronal arbor
Relative density (a.u.)
OT Layer Position (Superficial-Deep)
J
Type I
Type II Ipsi
Type II Contra
OT A-P axis
OT M-L axis
Arbor range (% OT)
Fish
Arbor A-P length (% OT)
Arbor M-L width (% OT)
pdf
10⁻³
Arbor A-P length
Arbor M-L width
K
Type I
Ipsilateral
r = 0.19, p = 0.8075
r = 0.41, p = 0.5882
r = -0.96, p = 0.0406
r = -0.60, p = 0.3972
r = -0.42, p = 0.5830
r = -0.29, p = 0.7071
r = 0.67, p = 0.3328
r = 0.23, p = 0.7715
r = 0.33, p = 0.6687
OT A-P (μm)
OT L-M (μm)
OT D-V (μm)
Soma A-P (μm)
Soma L-M (μm)
Soma D-V (μm)
L
Type II
Ipsilateral
Contralateral
r = -0.57, p = 0.0855
r = -0.75, p = 0.0126
r = 0.54, p = 0.1095
r = 0.23, p = 0.5276
r = 0.16, p = 0.6580
r = -0.19, p = 0.5966
r = 0.76, p = 0.0108
r = 0.61, p = 0.0585
r = -0.77, p = 0.0085
r = 0.50, p = 0.1412
r = 0.29, p = 0.4131
r = -0.64, p = 0.0481
r = -0.93, p = 0.0001
r = -0.71, p = 0.0226
r = 0.84, p = 0.0026
r = -0.58, p = 0.0758
r = -0.34, p = 0.3299
r = 0.58, p = 0.0814
OT A-P (μm)
OT L-M (μm)
OT D-V (μm)
Soma A-P (μm)
Soma L-M (μm)
Soma D-V (μm)

Figure S2: NI Type I and Type II neurons, related to Figure 2. (A) Et(*gata2a*:EGFP);Tg(*atoh7*:GFP) double transgenic used for electroporation of cells in the larval isthmus. Maximum intensity projection overlaid with registered *chata* expression. Scale bar, 50 μm. (A') Isthmic region showing cells of the locus coeruleus (LC) in close proximity to *chata* expression. Scale bar, 25 μm. (B,B') Electroporated neuron in the isthmic region projecting to the hypothalamus, labelled by *kctd15a*:GFP [S1]. (B') shows lateral view. Scale bars, 50 μm. (C) Photo-activated PVNs in the rostral OT of a Tg(*alpha tubulin*:C3PA-GFP) larva. White arrow indicates location of photo-activation that resulted in labelling of several PVN somata. White arrowheads mark a single axon projecting from a photo-activated PVN. Yellow asterisks mark photo-activated regions in additional specimens that resulted in labelling of tectofugal projections to the ventrolateral anterior hindbrain, whereas red asterisks mark regions which did not. White dotted line indicates midbrain-hindbrain boundary. Scale bar, 25 μm. (C') Terminal arbor (white arrow) of the axon in (C), located in the ventrolateral anterior hindbrain. Complete traced projection is shown in panel (D), Figure 2A and Video S1. (D) Registered tracing of Type I neuron (white) and photo-activated OT PVN (red), overlaid with relevant markers. Same example cells as Figure 2A. Scale bar, 50 μm. (D',D'') Magnified views of AF7 arborization and presumptive dendrite in hindbrain. (E) Registered tracing of Type II neuron (white), shown also in Figure 2B. (F) Type II axon decussating through the post-optic commissure (poc), marked by expression of *dbx1a*:Gal4 (red). The optic chiasm (oc), is located more rostrally and marked by *atoh7*:GFP (blue). Scale bar, 50 μm. Inset: Lateral view. (G-H) Registered tracings of Type I and Type II neurons, along with density profiles showing axon terminal distributions across tectal laminae. (I) Mean tectal lamination density profiles for all traced Type I ($n = 5$) and Type II ($n = 10$) cells. (J) Size and locations of Type I and Type II terminal arbors in OT. Bottom panel shows the distribution of arbor length and width as a fraction of tectal length/width. (K-L) Analysis of topographic innervation of OT. Locations of electroporated Type I ($n = 4$) and Type II ($n = 10$) cell bodies are plotted against centroid locations of terminal arbors in OT. We only considered datasets where there was no sign of registration error in any region of the brain and therefore one traced Type I cell was excluded due to registration uncertainty in the isthmic hindbrain. Linear fits to the data are shown with 95% confidence bounds. r, Pearson's correlation coefficient. Student's t-test.

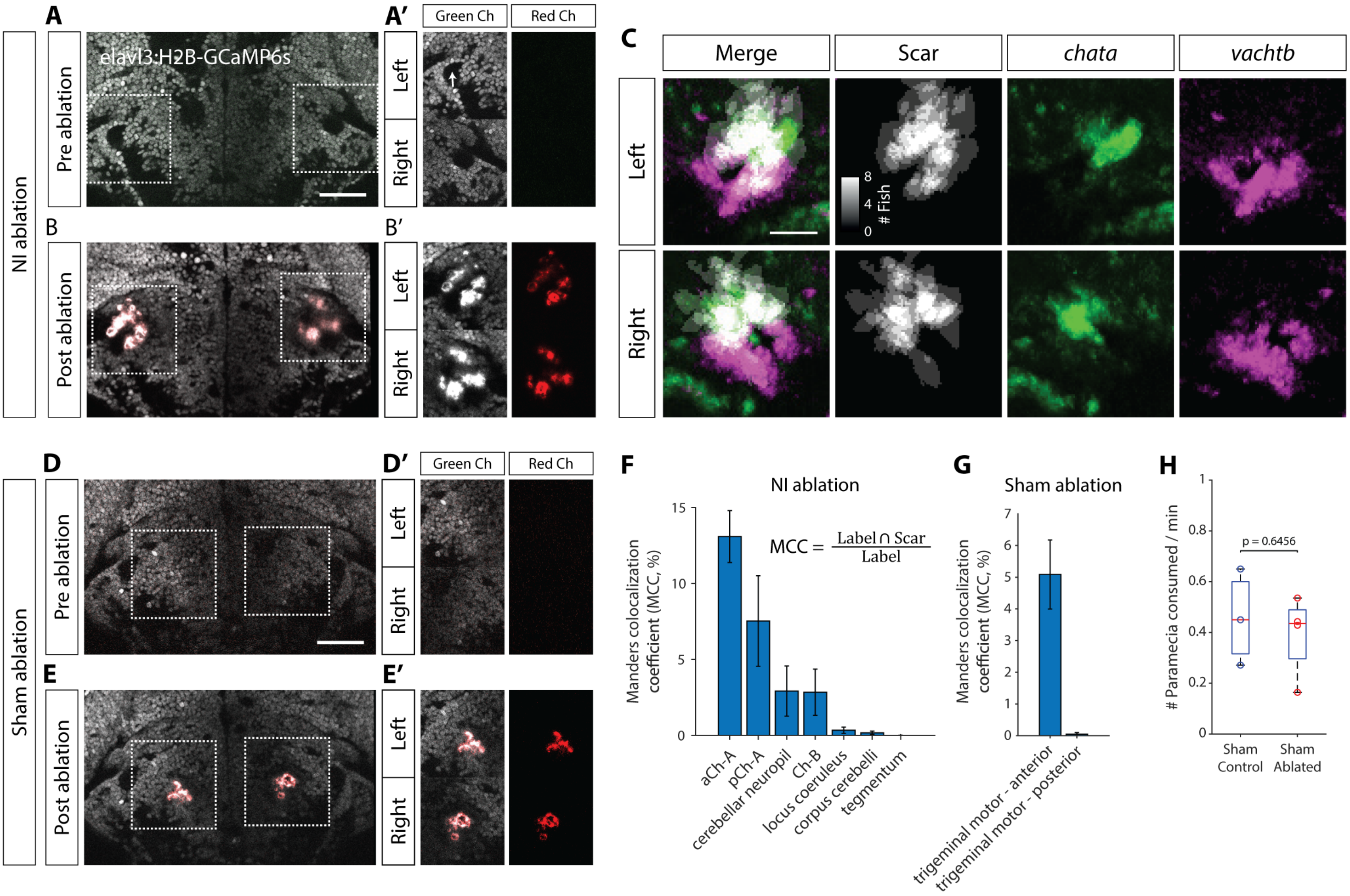

Figure S3: Laser ablations in isthmus, related to Figure 4. (A) 2-photon image of a 6 dpf Tg(*elavl3*:H2B-GCaMP6s) brain at the level of NI before ablation. Scale bar, 50 μm. (A') Isthmic region marked with dotted boxes in (A), showing green and red fluorescence channels. Before ablation there is no signal in the red channel. White arrow marks location of the cerebellar fascicle that can be used to locate NI. (B) Post-ablation image of the same brain. (B') The auto-fluorescent ablation scar can be seen in both green and red channels. (C) Sum of registered binary masks of ablation scars from 8 fish, overlaid with *chata* and *vachtb* expression. Scale bar, 25 μm. (D-E') Sham ablation of a region medially adjacent to NI. (F) Manders' colocalization coefficients (MCC) quantifying the percentage of voxels in ZBB brain regions that co-localize with the ablation scar for NI-ablations. Mean $\pm$ sem, $N = 8$ fish. (G) MCC quantification for sham ablation. (H) Prey consumption rates for sham-ablated larvae and their agarose-mounted sibling controls. Two sample t-test.

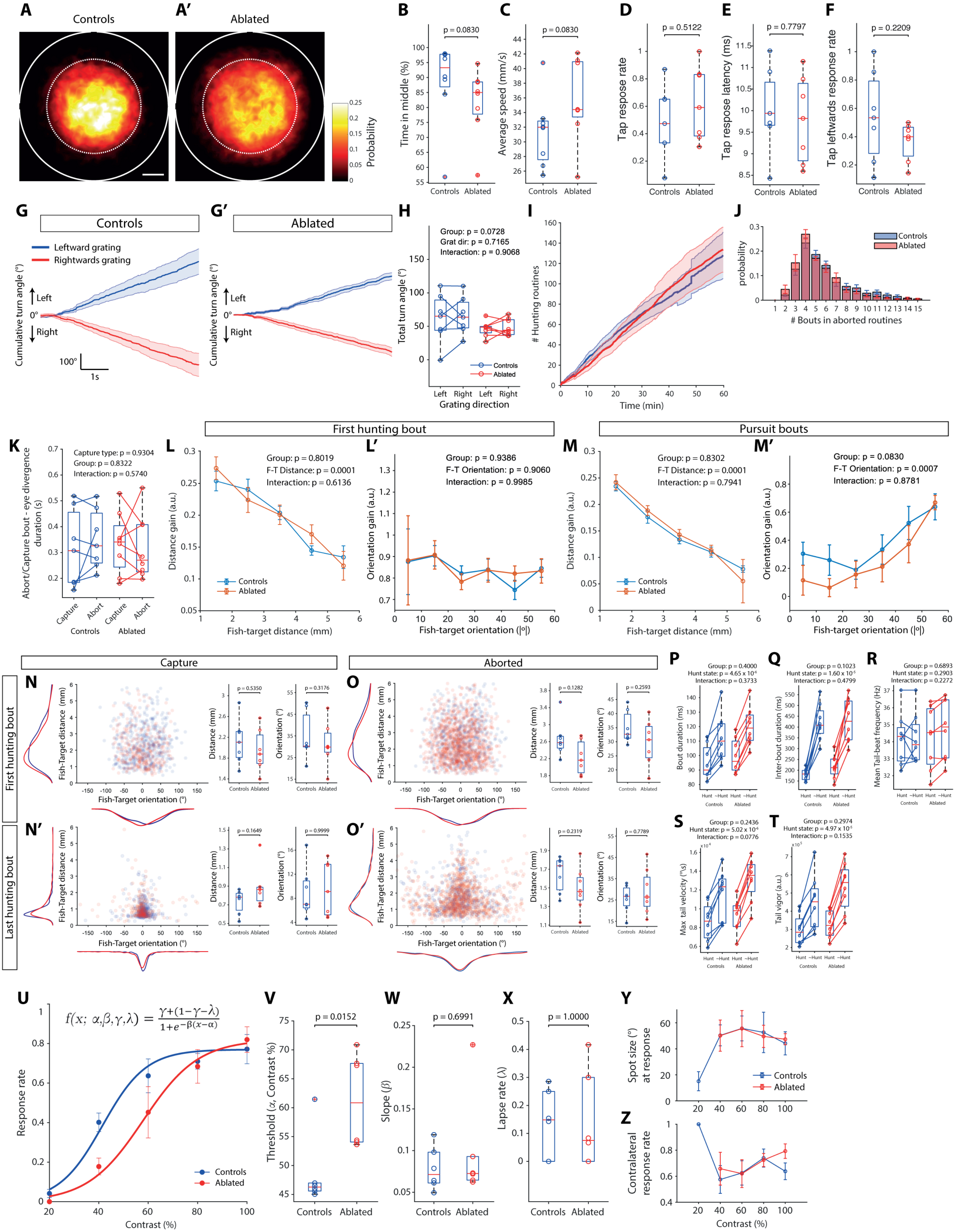

A
Controls
A'
Ablated
Probability
B
p = 0.0830
Time in middle (%)
C
p = 0.0830
Average speed (mm/s)
D
p = 0.5122
Tap respose rate
E
p = 0.7797
Tap respose latency (ms)
F
p = 0.2209
Tap leftwards response rate
Controls Ablated
G
Controls
Leftward grating
Rightwards grating
Cumulative turn angle (°)
Left
Right
100°
1s
G'
Ablated
H
Group: p = 0.0728
Grat dir: p = 0.7165
Interaction: p = 0.9068
Total turn angle (°)
Grating direction
I
Hunting routines
Time (min)
J
probability
Bouts in aborted routines
K
Capture type: p = 0.9304
Group: p = 0.8322
Interaction: p = 0.5740
Abort/Capture bout - eye divergence duration (s)
First hunting bout
L
Group: p = 0.8019
F-T Distance: p = 0.0001
Interaction: p = 0.6136
Distance gain (a.u.)
Fish-target distance (mm)
L'
Group: p = 0.9386
F-T Orientation: p = 0.9060
Interaction: p = 0.9985
Orientation gain (a.u.)
Fish-target orientation (|°|)
Pursuit bouts
M
Group: p = 0.8302
F-T Distance: p = 0.0001
Interaction: p = 0.7941
M'
Group: p = 0.0830
F-T Orientation: p = 0.0007
Interaction: p = 0.8781
Capture
Aborted
First hunting bout
Last hunting bout
N
Fish-Target distance (mm)
Fish-Target orientation (°)
p = 0.5350
p = 0.3176
Distance (mm)
Orientation (°)
O
p = 0.1282
p = 0.2593
N'
p = 0.1649
p = 0.9999
O'
p = 0.2319
p = 0.7789
P
Group: p = 0.4000
Hunt state: p = 4.65 x 10^-6
Interaction: p = 0.3733
Bout duration (ms)
Q
Group: p = 0.1023
Hunt state: p = 1.60 x 10^-5
Interaction: p = 0.4799
Inter-bout duration (ms)
R
Group: p = 0.6893
Hunt state: p = 0.2903
Interaction: p = 0.2272
Mean Tail-beat frequency (Hz)
S
Group: p = 0.2436
Hunt state: p = 5.02 x 10^-6
Interaction: p = 0.0776
Max tail velocity (°/s)
T
Group: p = 0.2974
Hunt state: p = 4.97 x 10^-5
Interaction: p = 0.1535
Tail vigor (a.u.)
Hunt ~Hunt Hunt ~Hunt
U
$f(x;\ \alpha,\beta,\gamma,\lambda) = \frac{\gamma+(1-\gamma-\lambda)}{1+e^{-\beta(x-\alpha)}}$
Response rate
Contrast (%)
V
p = 0.0152
Threshold (α, Contrast %)
W
p = 0.6991
Slope (β)
X
p = 1.0000
Lapse rate (λ)
Y
Spot size (°) at response
Z
Contralateral response rate
Contrast (%)

Figure S4: Behavior of NI-ablated larvae, related to Figure 4. (A,A') Distribution of control and NI-ablated larvae in the behavioral arena. Outer white circle marks edge of the arena and inner dotted circle shows the area in which larvae were considered 'in middle' for triggered presentation of visual stimuli. For each fish, a 2D probability histogram was generated for the duration of the experiment and convolved with a 2D gaussian filter (sigma = 15 px, 24.8 px/mm). Map shows mean probability density across fish. Scale bar, 5 mm. (B) Percentage of time larvae spent 'in middle' of the arena. (C) Average speed of the larvae over the duration of the experiment. (D-F) Response rate, latency and leftward response probability for mechano-acoustic 'tap'-evoked escapes. Data points are median values for each fish. Wilcoxon rank sum tests. (G-G') Cumulative turn angle in response to directional drifting gratings (mean $\pm$ sem). (H) Total cumulative turn angle during period of the grating stimulus. Two-way repeated measures ANOVA. (I) Cumulative number of hunting routines initiated by NI-ablated and control larvae over the course of the experiment. (J) Distribution of number of bouts within aborted hunting routines. (K) Time interval from end of capture swim or negative-gain (abort) bout to eye divergence. Data points are median per fish. Two-way repeated measures ANOVA. (L-M') Distance- and orientation-gain for first bout in hunting sequence and subsequent prey-tracking bouts. Data was binned based on prey orientation/distance. Mean $\pm$ sem of median values for each fish. Two-way ANOVA. (N-O') Spatial locations of target *Paramecia* at hunting initiation and immediately prior to capture swims (N') or at termination of aborted routines (O'). Data points and marginal histograms show data for control (blue) and ablated (red) larvae. Wilcoxon rank sum tests. (P-T) Motor kinematics for swim bouts within hunting routines ('Hunt') or when fish were not hunting ('$\sim$Hunt'). Data points represent median values for each fish. Two-way repeated measures ANOVA. (U) Response rates to looming stimuli of varying contrast. Curves show psychometric fits for control ($N = 6$) and NI-ablated ($N = 6$) groups. Note that 5 control and 4 ablated larvae were also tested for performance in hunting and other behaviors (Figure 4 and panels A-T) whereas the remaining larvae were tested only for loom-avoidance. (V-X) Quantification of psychometric fit parameters for individual fish. Wilcoxon rank sum tests. (Y) Angular size of looming spot at time of escape response. (Z) Frequency of escapes directed away from the location of the looming stimulus. Lack of data at 20% contrast in the NI-ablated group is due to the fact that no fish responded at this contrast level. Data presented as mean $\pm$ sem.

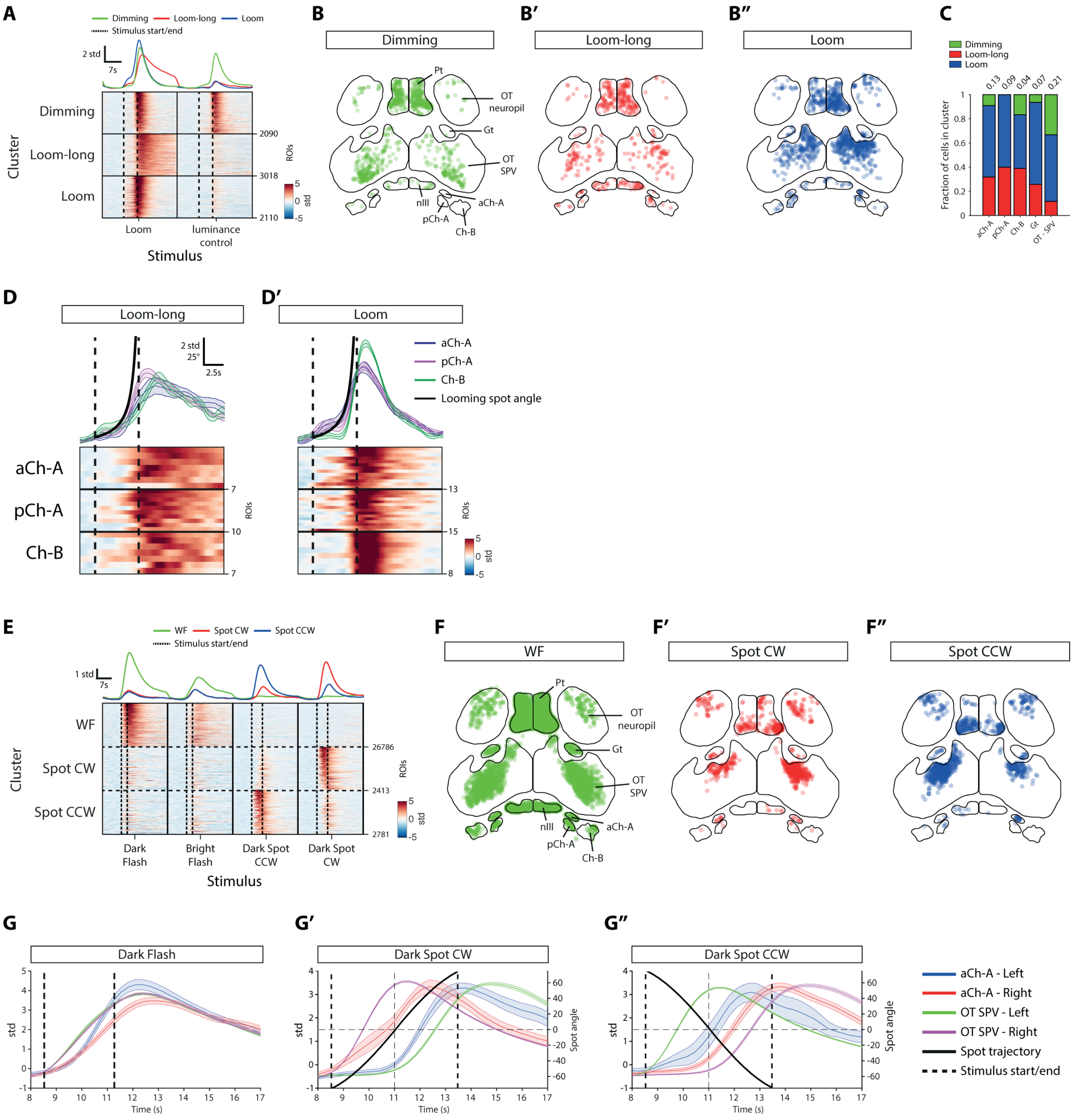

Figure S5: Visually evoked neural activity, related to Figure 5. (A) Clustered VRVs for the looming experiment. Top shows average responses of each cluster. Rows in the raster show VRVs for all clustered cells. The number of neurons in each cluster is shown on the right. (B-B″) Anatomical distribution of cells assigned to visual clusters in the looming experiment. (C) Visual cluster assignments for each brain area. Numbers on top show the fraction of cells in the brain region that were assigned cluster identities. (D,D′) Responses of NI neurons in the Loom and Loom-long clusters. (E) All clustered VRVs for hunting experiment. (F-F″) Locations of visually responsive cells in the hunting experiment. (G-G″) Fluorescent time-series of aCh-A and OT-SPV neurons to prey-like visual stimuli (mean ± sem). Dashed lines indicate period of stimulus presentation and horizontal and vertical dotted lines indicate when the moving spot is at the centre of the screen (directly ahead of the fish). Negative spot angles correspond to the left visual hemifield. nIII, oculomotor nuclei; OT SPV, optic tectum stratum periventriculare; Gt, griseum tectale; Pt, pretectum.

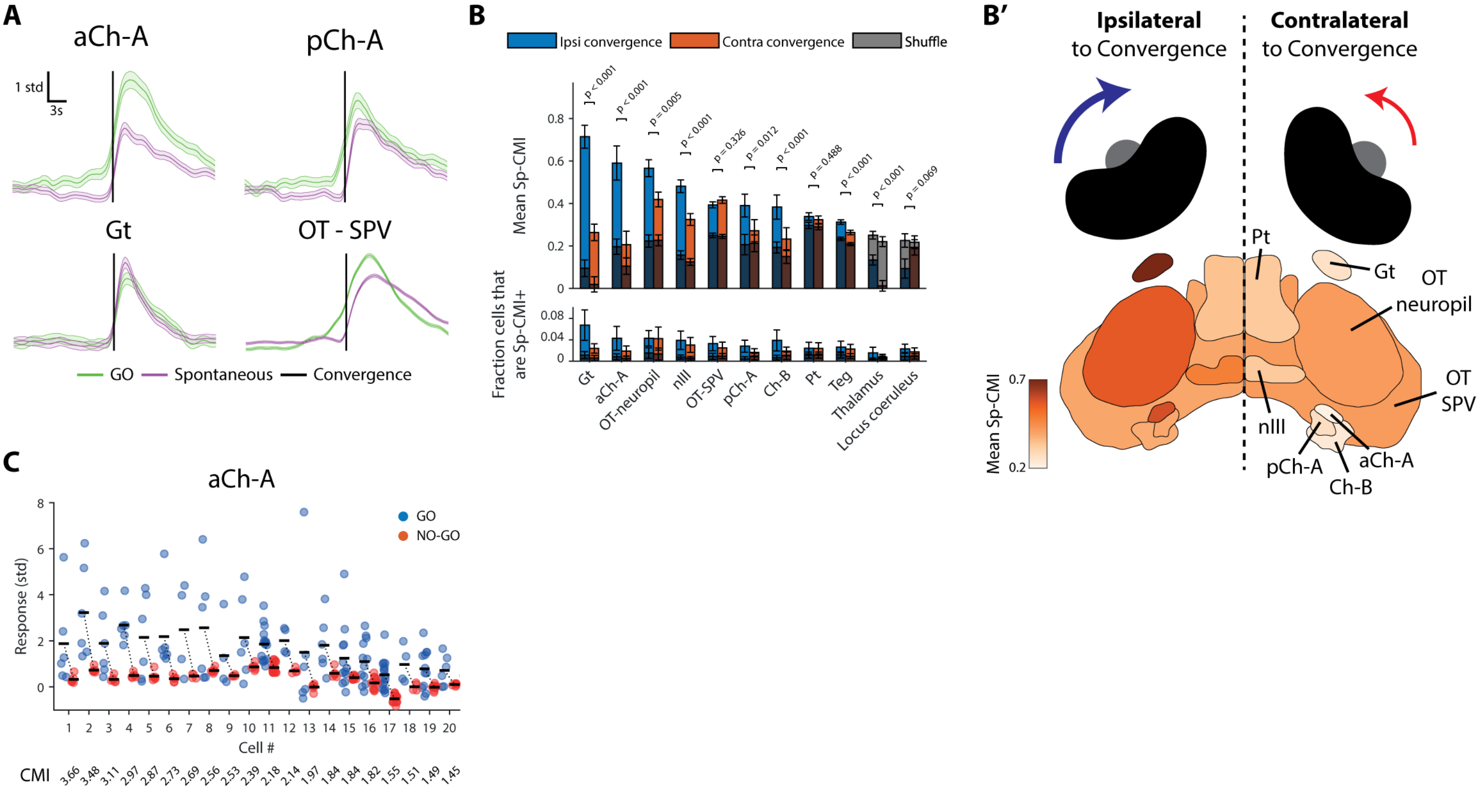

Figure S6: Convergence-related neural activity, related to Figure 6. (A) Average normalized fluorescence response of aCh-A, pCh-A, Gt and OT-SPV neurons triggered on time of convergent saccades (mean ± sem). (B,B') Sp-CMI scores. Mean Sp-CMI and fraction of Sp-CMI+ cells for several imaged brain regions. Mean ± sem. Wilcoxon signed rank tests between each region's ipsilateral and contralateral convergence distributions. (B') shows brain areas colored by mean Sp-CMI scores for ipsilateral and contralateral convergences. (C) Responses of individual aCh-A cells across the complete set of GO and NO-GO trials during which their calcium activity was recorded. Twenty cells with varying CMI scores are displayed, sorted by descending CMI.

Supplemental Reference

S1. Heffer, A., Marquart, G.D., Aquilina-Beck, A., Saleem, N., Burgess, H.A., and Dawid, I.B. (2017). Generation and characterization of Kctd15 mutations in zebrafish. PloS One 12, e0189162.
