# Supplementary material for: Nucleus Isthmi Is Required to Sustain Target Pursuit during Visually Guided Prey-Catching
Source: Curr Biol. 2019 Jun 3;29(11):1771–1786.e5. doi: 10.1016/j.cub.2019.04.064 (PMC6557330; doi:10.1016/j.cub.2019.04.064)
Supplement: Document S2. Article plus Supplemental Information [file mmc4.pdf]

Article

Current Biology

Nucleus Isthmi Is Required to Sustain Target Pursuit during Visually Guided Prey-Catching

Graphical Abstract

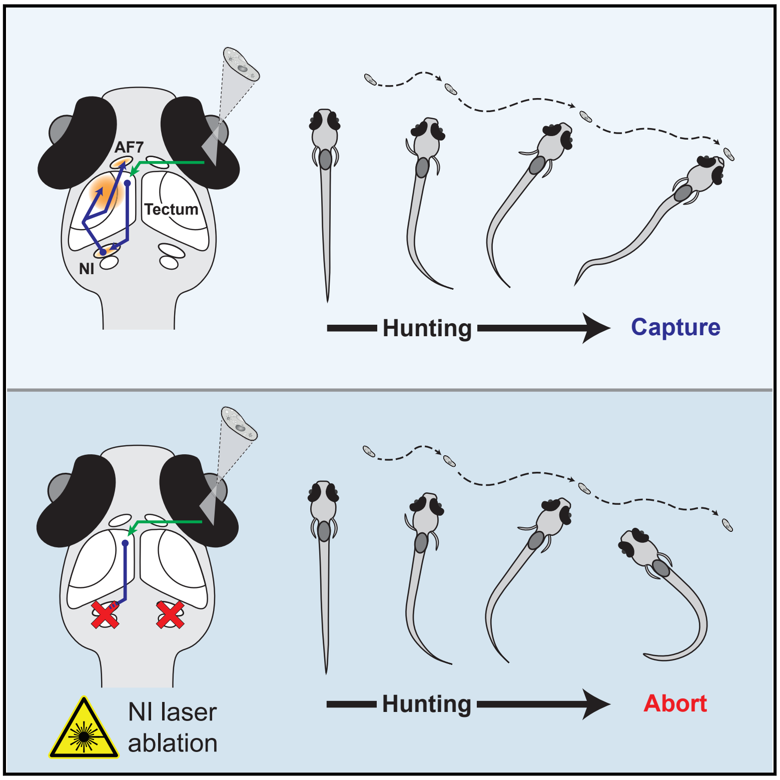

Highlights

- Nucleus isthmi contains two types of neuron with distinct (pre)-tectal connectivity
- Neural activity in nucleus isthmi is recruited at onset of hunting behavior
- Nucleus isthmi is required for maintenance, but not initiation, of hunting routines

Authors

Pedro M. Henriques, Niloy Rahman, Samuel E. Jackson, Isaac H. Bianco

Correspondence

i.bianco@ucl.ac.uk

In Brief

Henriques et al. identify two classes of projection neuron connecting the nucleus isthmi (NI) to the optic tectum and pretectum. Functional calcium imaging and laser ablation combined with behavioral analyses indicate that NI is recruited during hunting and functions to sustain prey-tracking sequences.

Henriques et al., 2019, Current Biology *29*, 1771–1786
June 3, 2019 © 2019 The Author(s). Published by Elsevier Ltd.
https://doi.org/10.1016/j.cub.2019.04.064

CellPress

CellPress

Nucleus Isthmi Is Required to Sustain Target Pursuit during Visually Guided Prey-Catching

Pedro M. Henriques,[1] Niloy Rahman,[1] Samuel E. Jackson,[1] and Isaac H. Bianco[1,2,*]
[1]Department of Neuroscience, Physiology and Pharmacology, University College London, London WC1E 6BT, UK
[2]Lead Contact
*Correspondence: i.bianco@ucl.ac.uk
https://doi.org/10.1016/j.cub.2019.04.064

SUMMARY

Animals must frequently perform a sequence of behaviors to achieve a specific goal. However, the neural mechanisms that promote the continuation and completion of such action sequences are not well understood. Here, we characterize the anatomy, physiology, and function of the nucleus isthmi (NI), a cholinergic nucleus thought to modulate tectal-dependent, goal-directed behaviors. We find that the larval zebrafish NI establishes reciprocal connectivity with the optic tectum and identify two distinct types of isthmic projection neuron that either connect ipsilaterally to retinorecipient laminae of the tectum and pretectum or bilaterally to both tectal hemispheres. Laser ablation of NI caused highly specific deficits in tectally mediated loom-avoidance and prey-catching behavior. In the context of hunting, NI ablation did not affect prey detection or hunting initiation but resulted in larvae failing to sustain prey-tracking sequences and aborting their hunting routines. Moreover, calcium imaging revealed elevated neural activity in NI following onset of hunting behavior. We propose a model in which NI provides state-dependent feedback facilitation to the optic tectum and pretectum to potentiate neural activity and increase the probability of consecutive prey-tracking maneuvers during hunting sequences.

INTRODUCTION

To accomplish a behavioral goal, it is often necessary for an animal to execute a sequence of component actions. Studies in various species have examined the organization and sequencing of behavioral elements in specific contexts [1]. Progress has been made identifying neural circuits involved in inducing specific behaviors, modulating features of behavior, and controlling the timing and serial execution of motor sequences (e.g., [2, 3]). However, little is known about neural mechanisms that explicitly maintain a behavioral state so as to facilitate the progression of action sequences to support task completion. In classic neuroethology, ‘‘stimulus-response chains’’ have been proposed in which a stereotyped sequence of sensorimotor transformations occurs because the outputs from one action act as the ‘‘releasing stimulus’’ for the next [4, 5]. However, for complex behaviors, such as those involving interactions between animals, the sequence of sensory inputs might be less reliable. In the context of hunting motile prey, the sensory circumstances and desired action at any time are determined by a combination of the actions of both predator and prey. Additionally, the presence of multiple prey might present competing stimuli that challenge the predator to sustain attention at a specific target.

Zebrafish perform visually guided hunting to capture motile prey [6, 7]. At larval stages, zebrafish swim in discrete swim bouts, separated by brief pauses, and hunting routines involve sequences of particular bout types that enable the animal to pursue, approach, and eventually strike at its target [8–11]. The optic tectum (OT), known as the superior colliculus (SC) in mammals, is well understood to be an important sensorimotor center involved in orienting and avoidance behaviors across vertebrates [12] and is central to visually guided hunting in zebrafish [7, 13]. In addition to receiving a substantial retinal input, OT and SC is interconnected with numerous other brain regions, including circuits thought to monitor and modulate tectal activity. One such structure is the nucleus isthmi (NI), thought to correspond to the parabigeminal nucleus (PBg) in mammals. These paired cholinergic nuclei are located at the midbrain-hindbrain boundary of the tegmentum and are reciprocally connected with OT and SC in all vertebrates studied [14, 15]. The NI and PBg has been described a satellite system of OT and SC and is a good candidate to modulate tectal activity during hunting. NI and PBg has been implicated in visual prey detection, tracking of moving targets, generation of binocular visual responses in rostral OT, and mechanisms of selective spatial attention (reviewed in [15]).

We first characterize the anatomy and connectivity of NI in larval zebrafish, identifying two distinct types of cholinergic projection neuron that connect either ipsilaterally or bilaterally to OT. Laser ablation of NI produced highly specific deficits in tectally mediated hunting and loom avoidance. Ablated larvae showed reduced contrast sensitivity to looming threats, suggesting that NI modulates either sensory detection or sensorimotor coupling in the context of this avoidance behavior. By contrast, during hunting, NI was not required for initiation of hunting routines or for detection or accurate targeting of prey. Instead, NI was specifically required to sustain hunting state comprising sequential prey-tracking maneuvers. By using a virtual hunting assay combined with calcium imaging, we found that NI activity was recruited at the onset of hunting behavior. Our results support a model in which NI provides state-dependent feedback facilitation to OT and/or pretectum during hunting to promote the progression of prey-tracking sequences. To our knowledge, this is

This is an open access article under the CC BY license (http://creativecommons.org/licenses/by/4.0/).

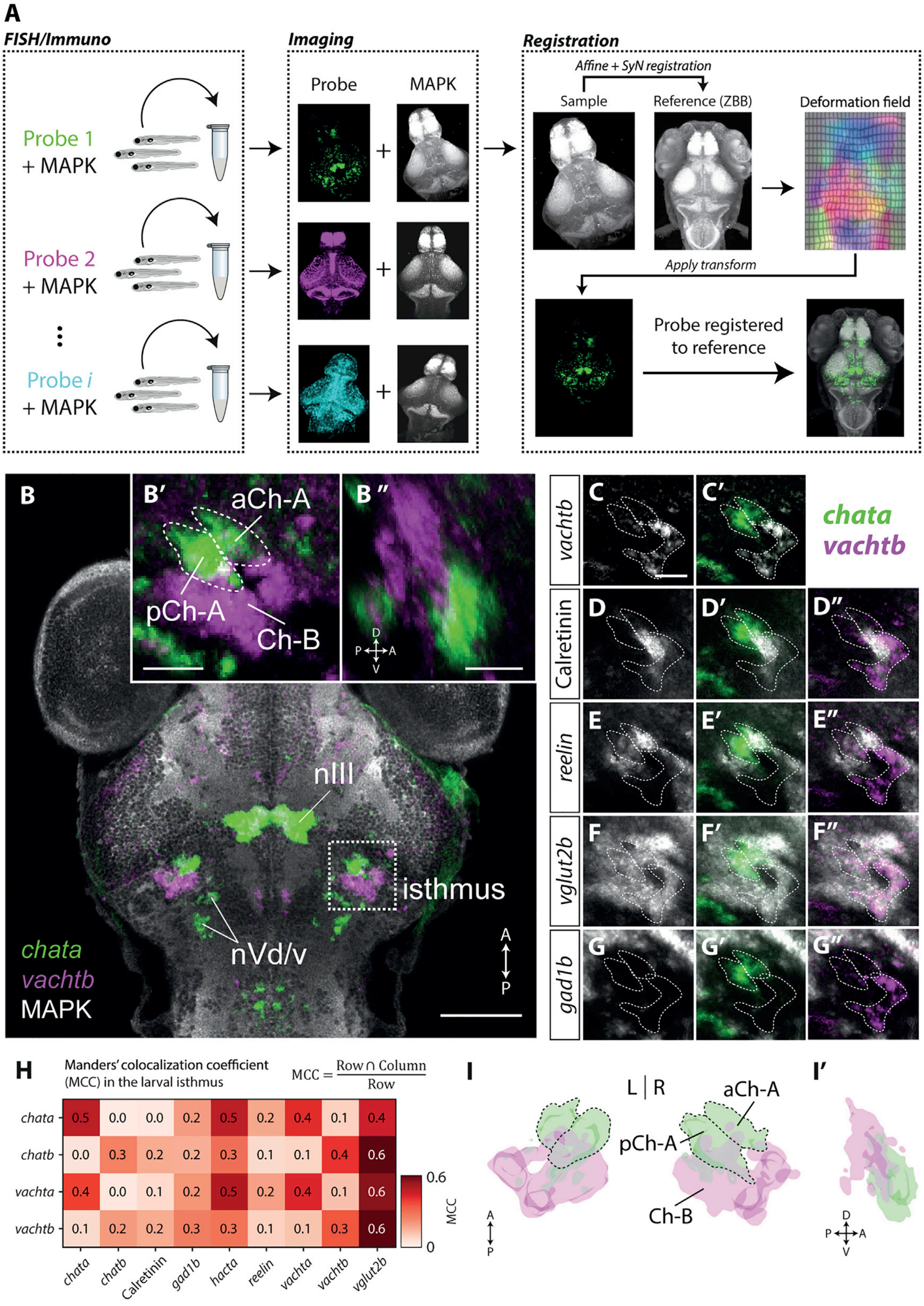

Figure 1. The Larval Zebrafish Isthmus Contains Two Cholinergic Gene Expression Domains

(A) Whole-brain registration pipeline. Fluorescent *in situ* hybridization for a target mRNA (probe) is combined with immunohistochemistry for MAPK on a separate fluorescence channel. Brain volumes are then imaged using 2-photon or confocal microscopy. For each brain, MAPK volumetric data ("sample") are registered to

(legend continued on next page)

one of the first descriptions of a neural circuit specifically required to maintain a sensorimotor action sequence.

RESULTS

The Isthmus Comprises Two Cholinergic Domains

We first sought to define the anatomical location, gene expression patterns, and connectivity of NI at late larval stages when zebrafish readily engage in innate prey-catching and loom-avoidance behaviors. Because expression of cholinergic markers is a defining feature of NI across vertebrates [14], we examined expression of genes encoding proteins required for synthesis (*chat*) and vesicular transport (*vacht*) of acetylcholine, which defines cholinergic neurons [16]. Zebrafish have two "cholinergic gene loci" (CGLa and CGLb), each encoding *chat* and *vacht* homologs with distinct expression patterns [17]. We examined expression in 6 days post-fertilization (dpf) larval brains, which were additionally immunostained for mitogen-activated protein kinase (MAPK), allowing fluorescent datasets to be registered to the "ZBB" brain atlas (Figure 1A) [18].

In agreement with [17], genes *within* each of the CGLa and CGLb loci showed very similar expression patterns across the brain, which we refer to as Ch-A (*chata*/*vachta*) and Ch-B (*chatb*/*vachtb*). By contrast, expression of CGLa versus CGLb genes was largely non-overlapping (Figures 1B and S1C–S1F).

Within the isthmic region, Ch-A and Ch-B expression was observed in two adjacent but largely non-overlapping domains, with Ch-B expression (221 ± 6 cells; n = 2) located lateral and dorsal to Ch-A expression (179 ± 3 cells; n = 4; Figures 1B and 1H). Double fluorescence *in situ* hybridization (FISH) for *chata* and *vachtb* directly confirmed these *in silico* expression localization results (Figure S1L). We also noted that the Ch-A domain could be subdivided into anterior (aCh-A) and posterior (pCh-A) subregions, with the location of the boundary marked by a large fascicle from the cerebellum (Figure S1B). Both Ch-A and Ch-B largely colocalized with *vglut2b* expression, but not with *gad1b* (Figures 1F–1H).

To distinguish NI from other tegmental cholinergic nuclei, namely the secondary gustatory-general viscerosensory nucleus (SGN/V) that is caudally adjacent to NI in adult zebrafish [19], we examined expression of additional markers. Although Calretinin has been shown to be expressed in the adult zebrafish SGN, but not NI [20], *reelin* has been shown to label NI at both adult [21] and early larval [22] stages. We found that Calretinin expression overlapped with part of the Ch-B domain, but not Ch-A (Figures 1D and 1H). By contrast, *reelin* expression colocalized with the Ch-A domain (Figures 1E and 1H).

In summary, we identified two domains of cholinergic gene expression in the larval zebrafish isthmus. The Ch-A domain likely corresponds to the larval NI and can be subdivided into anterior (aCh-A) and posterior (pCh-A) regions. At least part of the SGN/V appears to express CGLb genes (Ch-B region) and is located dorso-laterally to the Ch-A domain (Figure 1I).

Two Types of NI Neuron with Distinct Patterns of Tectal and Pretectal Connectivity

A defining feature of NI is reciprocal connectivity with OT [12]. We used focal electroporation, lipophilic dye tracing, and photo-activatable fluorescent proteins to examine NI-OT interconnectivity in 6 dpf larval zebrafish.

We first used focal electroporation of fluorescent dextran conjugates to examine the morphology and projection patterns of single neurons [23]. We targeted cells in the isthmic region at 4 dpf, using double transgenic Et(*gata2a*:EGFP);Tg(*atoh7*:GFP) larvae (Figure S2A), and imaged labeled cells at 6 dpf. Et(*gata2a*:EGFP) expression in the locus coeruleus, which lies in close proximity to NI (Figure S2A) [21], helped us target isthmic neurons for electroporation, and *atoh7*:GFP labeling of retinal ganglion cells (RGCs) and their axons allowed us to localize the axon terminals of electroporated neurons with respect to tectal laminae and other retinal arborization fields. The transgenic labeling was further used to register imaging data to the brain atlas, allowing us to estimate the location of electroporated somata with respect to cholinergic expression domains. By re-imaging individual brains, we established that this method provided a localization accuracy of 2.7 ± 1 μm (n = 11 cells; STAR Methods), approximately half the diameter of a neuronal cell body (≈6 μm).

In total, we labeled 21 neurons in the isthmic region, which had two major efferent targets. Four cells projected to the ipsilateral hypothalamus (Figure S2B), and their somata were mostly located within the dorso-lateral Ch-B domain (Figure 2F). Because the lateral hypothalamus is the established target of SGN/V [24], we conclude that these cells are larval SGN/V neurons.

We identified two types of isthmic neuron that projected to OT, which displayed unique neurite morphologies and projection profiles. The axons of type I cells (n = 7) innervated both the ipsilateral pretectal arborization field 7 (AF7) and OT (Figures 2A, S2D, and S2G; Video S1). After coursing rostrally, possibly through the isthmo-pretectal tract, type I axons densely innervated AF7 and additionally extended a collateral into anterior OT. Terminal axon arbors within OT extended over a limited area (length: 28% ± 3% of anterior–posterior (A-P) axis; width:

the MAPK "reference" volume of ZBB. The computed deformation field is then applied to the probe channel to transform it into reference space, allowing *in silico* comparison of multiple expression patterns.

(B) Dorsal view showing expression of the cholinergic genes *chata* and *vachtb* across the brain. Each pattern represents a median of multiple registered single brains (n = 4; both) superimposed on the MAPK reference. Images show a maximum-intensity projection through focal planes encompassing the isthmus (90 μm). Scale bar, 100 μm. (B' and B'') Enlarged view of isthmic region shows segregation of Ch-A and Ch-B domains. Scale bars, 25 μm.

(C–G'') *In silico* colocalization of molecular markers in the isthmus: *vachtb* (C and C'), Calretinin (D and D''), *reelin* (E and E''), *vglut2b* (F and F''), *gad1b* (G and G''). Dotted boundaries delineate aCh-A, pCh-A, and Ch-B regions. Images show the same single focal plane. In each row, the indicated marker is shown in gray and *chata* and *vachtb* are overlaid in green and magenta, respectively. Scale bar, 25 μm.

(H) Mander's colocalization coefficient (MCC) quantifying overlap of markers in the larval isthmus. For each row marker, MCC represents the fraction of labeled voxels that coexpress the column marker. Values are means of pairwise comparisons across all specimens (Figure S1A).

(I) 3D rendering of cholinergic expression domains in the isthmus. (I') Sagittal view of 3D rendering shows right isthmic region.

nIII, oculomotor nucleus; nVd/v, dorsal and ventral trigeminal nuclei. See also Figure S1.

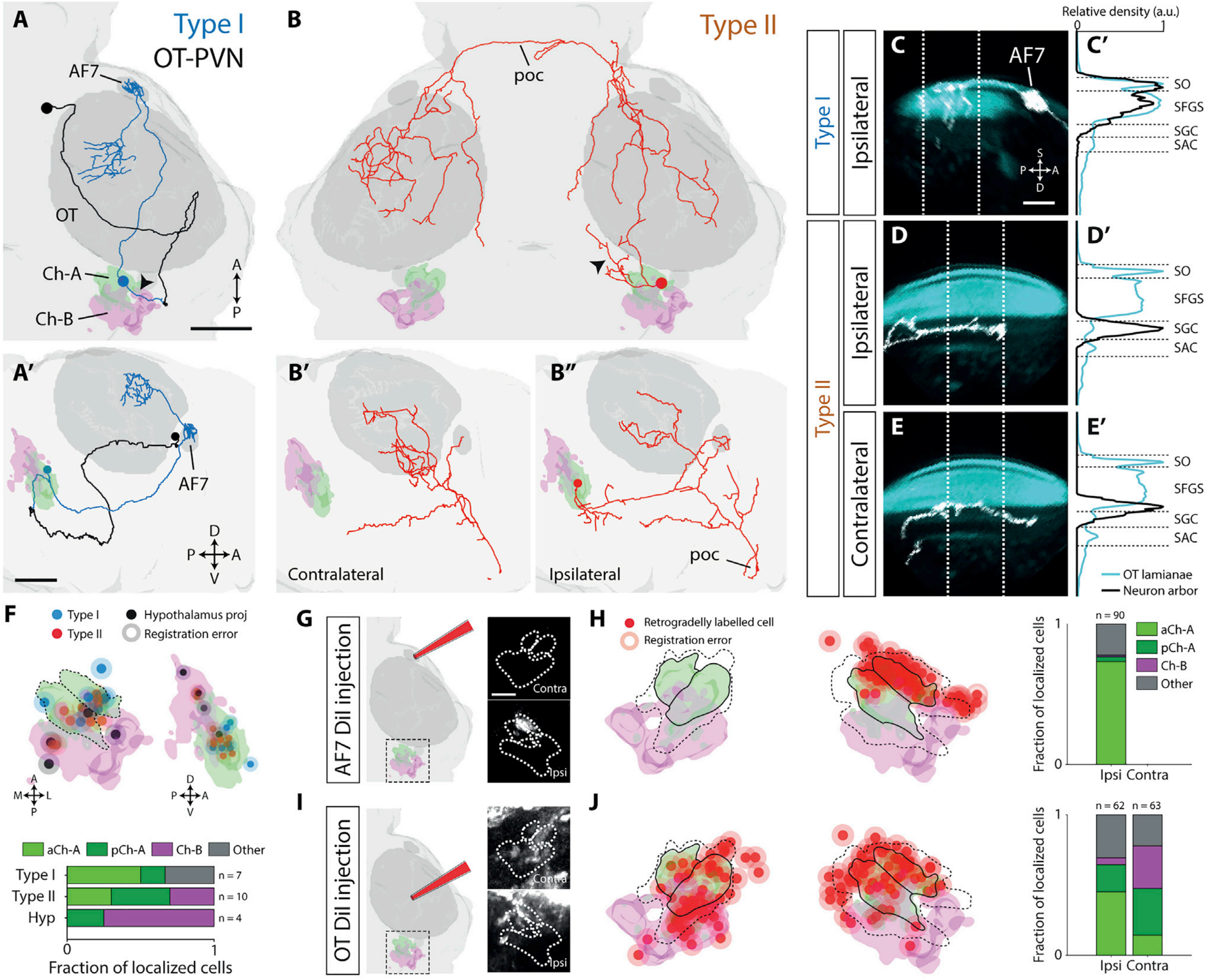

Figure 2. Two Types of NI Projection Neuron with Distinct Tectal and Pretectal Connectivity

(A and B) Examples of a type I (A and A') and a type II (B–B") projection neuron that were labeled by electroporation. (A) also shows a photo-activated OT PVN neuron, projecting to isthmus (black). Neuronal tracings have been registered to the atlas and are overlaid with 3D-rendered masks displaying relevant brain areas. Top panels are dorsal views, and lower panels show lateral views. Black arrowheads indicate the putative dendrites of isthmic neurons. Scale bars, 50 μm.

(C–E') Type I (C and C') and type II (D–E') axonal arborizations (white) in OT neuropil laminae (cyan). The imaging volume was rotated clockwise about the A-P axis ~30° and then clockwise about the left-right (L-R) axis ~40° to enable clearer visualization of the laminae. A density projection (C', D', and E') along the superficial-deep tectal axis was taken over a volume that encompasses most of the axon arbors (60 μm). Dotted white lines delineate the area used to compute density profiles shown on the right (normalized to maximum). Scale bar, 25 μm.

(F) Localization of electroporated cells within isthmic domains. Top shows dorsal and lateral renderings of the Ch-A and Ch-B domains with registered locations of electroporated somata. Shading around each point represents mean registration uncertainty. Bottom quantifies localization of cell types across domains.

(G) Iontophoretic labeling of AF7. Schematic of DiI application is shown with insets showing the isthmus of a single registered brain with retrograde labeling in the ipsilateral aCh-A domain.

(H) Locations of retrogradely labeled somata following iontophoretic labeling of the right AF7 (n = 6 fish). Black lines show mean boundaries of aCh-A and pCh-A, and dotted lines show Ch-A maximum boundaries.

(I) Iontophoretic labeling of OT neuropil. Insets show retrograde labeling in both the ipsilateral and contralateral isthmus.

(J) Locations of labeled somata following iontophoretic labeling of the right OT neuropil (n = 10 fish).

A, anterior; AF7, arborization field 7; D, deep; OT, optic tectum; P, posterior; poc, post-optic commissure; S, superficial. See also Figure S2 and Videos S1 and S2.

28% ± 2% of M-L axis; Figure S2J) and were preferentially localized within the anterior portion of the tectum (Figure S2J). Type I cells innervated superficial retinorecipient laminae of OT (*stratum opticum* [SO] and *stratum fibrosum et griseum* [SFGS] layers; Figures 2C, S2G, and S2I). These neurons also extended a large neurite, which we hypothesize is the primary dendrite, posterolaterally from the soma, terminating in a dense arbor in the lateral hindbrain (Figures 2A, black arrowhead, and S2D"). Registration localized most type I somata within, or just adjacent to, the Ch-A domain (Figure 2F).

In contrast, type II neurons (n = 10) projected bilaterally, innervating both tectal hemispheres (Figures 2B and S2H; Video S2). Their axons decussated via the post-optic commissure (Figure S2F), and axon terminals extended more broadly across the tecta than type I cells (ipsi length: 42% ± 4%, width: 64% ± 5%; contra length: 43% ± 5%, width: 56% ± 5%; Figure S2J). Terminal arbors showed some variation in laminar targeting but were generally localized to deeper tectal layers than type I axons (SFGS, *stratum griseum centrale* [SGC], and *stratum album centrale* [SAC]; ipsilateral arbors also extended into more superficial layers; Figures 2D, 2E, S2H, and S2I). In contrast to type I cells, the presumptive dendrites of type II neurons extended in a rostro-medial direction toward the mesencephalic tegmentum (Figure 2B, black arrowhead). Volumetric registration placed the majority of type II somata within the Ch-A domain with a few cells assigned to the Ch-B region (Figure 2F).

We took advantage of the distinct efferent targets of type I and type II cells to retrogradely label larger numbers of somata in the isthmus. Using iontophoresis, we focally applied DiI to either AF7 or the tectal neuropil of fixed Tg(*elavl3*:H2B-GCaMP6s) brains. Following dye application to AF7, retrogradely labeled somata in the isthmic region were almost exclusively localized to the ipsilateral aCh-A region (Figures 2G and 2H). By contrast, DiI application to the tectal neuropil labeled isthmic neurons in Ch-A and Ch-B regions (Figures 2I and 2J). Because only type II cells innervate contralateral OT, we reasoned that somata contralateral to the dye application site represent the isthmic location of this cell type (Figure 2J). Thus, we conclude that, although type I cells are almost exclusively localized to the ipsilateral aCh-A region, type II cells are found in aCh-A, pCh-A, and the anterior portion of Ch-B.

Because NI-OT projections are topographically mapped in several species [25–28], including teleosts [29], we asked whether this was also true for zebrafish larvae. We compared the locations of electroporated cell bodies to the centroid of their axon terminal arbors within OT. Our data suggest a topographic relationship may exist between the dorsoventral position of type I somata (n = 4) and the anterior-posterior position of their axon arbors in ipsilateral OT (Figure S2K), albeit with a limited sample size. Type II neurons (n = 10) showed clear topography in their ipsilateral OT projections, such that the anterior-dorsal-medial tectum is innervated by the posterior-dorsal-medial isthmus (Figure S2L). On the contralateral side, however, topography was less conspicuous.

The Tectum Projects to Ipsilateral NI

To establish whether OT projects to the isthmus, we performed anterograde tracing using the photo-activatable fluorescent protein PA-GFP [30]. Using a 2-photon microscope, we photo-activated a small number of tectal periventricular neurons (PVNs) at 5 dpf in Tg(*alpha tubulin*:C3PA-GFP) larvae and imaged labeled projections at 6 dpf.

When PVNs in rostral OT were labeled, we consistently observed axonal projections to the ventrolateral anterior hindbrain (n = 4; Figure S2C). These projections terminated in close proximity to the dendritic arbors of type I neurons (Figures 2A and S2D; Video S1). By contrast, photo-activation of caudal OT sites did not reveal tecto-isthmic projections (n = 5; Figure S2C, red asterisks).

Taken together, our data support the conclusion that there exist two types of cholinergic NI projection neurons in larval zebrafish. NI type I cells are localized to the anterior portion of the Ch-A expression domain, project ipsilaterally to the pretectal AF7 arborization field and rostral OT, and likely receive reciprocal afferent input from rostral ipsilateral OT. Type II cells are putatively a second type of NI neuron. They are located in the Ch-A and anterior Ch-B region and bilaterally innervate the deeper laminae of both optic tecta.

High-Speed Tracking of Hunting Sequences

Zebrafish larvae preferentially respond to prey within their anterior visual field [9, 31], represented by the rostral portion of OT [32]. Furthermore, AF7 is preferentially innervated by the temporal retina, responds strongly to prey-like visual cues, and its ablation impairs hunting [33, 34]. Thus, the fact that NI type I cells innervate both these regions is highly suggestive of a role in modulating tectally mediated predatory behavior.

Zebrafish initiate hunting by performing a convergent saccade that greatly expands their binocular visual field, and elevated ocular vergence is maintained throughout the hunting routine [9]. After orienting toward and approaching their prey using a sequence of discrete turns ("J-turns" and "HAT" turns) and forward swim bouts, larvae position their prey within a strike zone before executing a kinematically distinct capture swim or "suction" to attempt to consume the prey [9, 11, 31, 35].

To investigate a role for NI in hunting, we developed a platform for high-speed automated tracking of larval behavior and predator-prey interactions. Individual fish swam freely within a behavioral arena and were provided with *Paramecia* to feed upon (Figure 3A). Eye and tail kinematics were extracted online at 700 Hz, and prey were automatically tracked at slower speeds. The onset and duration of hunting routines were automatically defined based on periods of elevated ocular vergence. A semi-automated procedure was used to annotate specific features and outcomes of individual routines. This included labels indicating which *Paramecium* the larva targeted, the nature of the final capture attempt (strike or suction), and whether that attempt was successful or not. To identify the target, we used a principled and conservative criterion. For each swim bout within a routine, we computed the distance-gain and orientation-gain as the fraction of distance or orientation (azimuth) between fish and prey that was eliminated by that bout (Figure 3C; STAR Methods). A *Paramecium* was designated as a target if it was initially located within the reactive perceptive field ($\leq$6 mm and within ±120°) [9, 31] and the distance-gain and orientation-gain for the first two bouts of the hunting sequence were positive with respect to that prey item (i.e., repeatedly orienting toward and approaching the prey; STAR Methods).

We observed that more than half of all hunting routines did not progress as far as the final attempt to consume prey but instead were prematurely aborted (60% ± 4%; n = 7 fish [784 routines]; Figures 3E and 3G) [36]. When we examined prey-tracking performance, we found that these aborted routines frequently incorporated swim bouts having negative orientation-gain *and* distance-gain, indicating that the larva was failing to pursue its prey. These negative-gain bouts occurred in 37% of aborted routines but only 9% of routines ending in a capture attempt. Moreover, in only ~3% of routines did larvae resume target

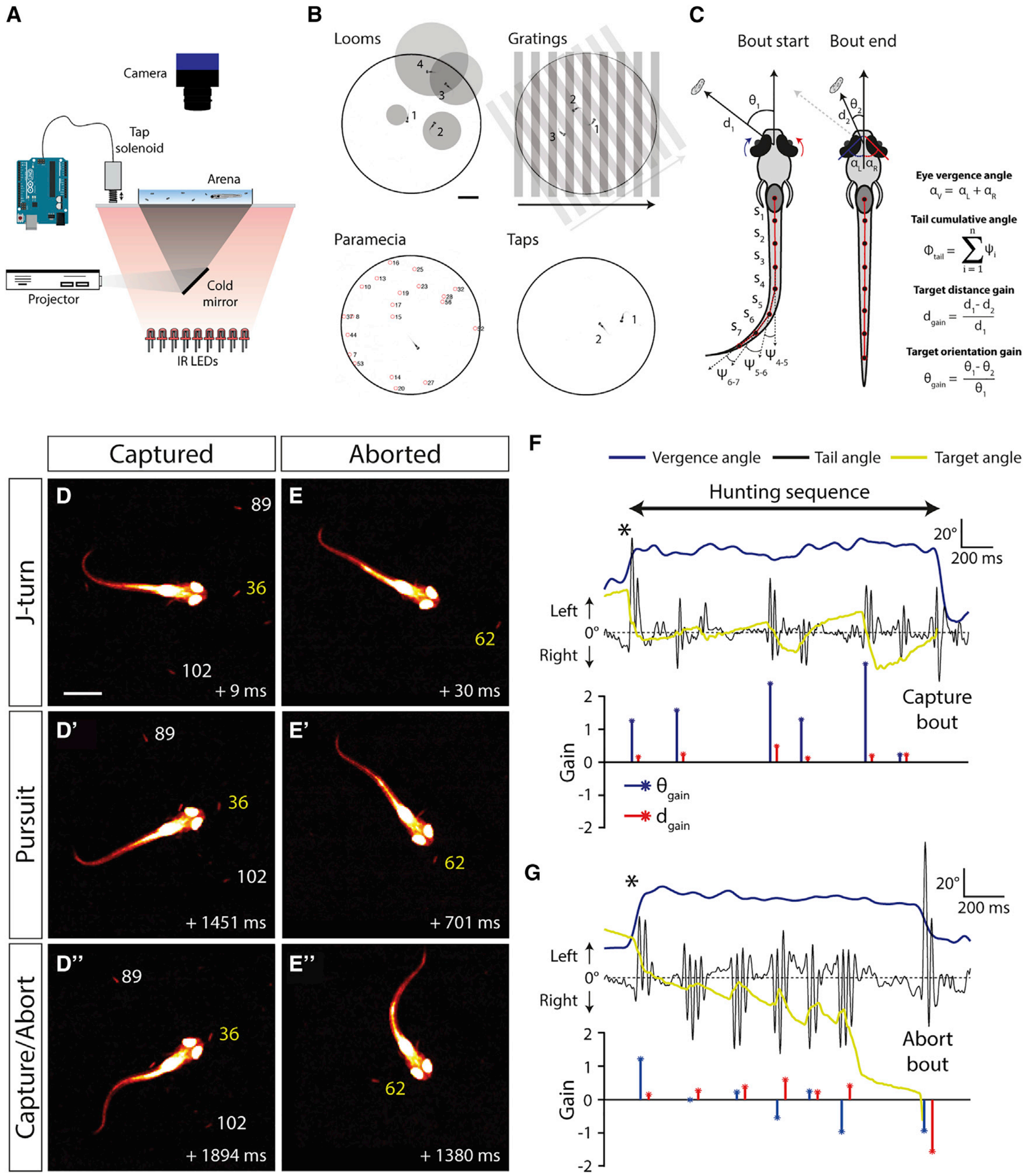

Figure 3. High-Speed Behavioral Tracking of Hunting Routines

(A) Experimental setup used for testing free-swimming behavior.

(B) Illustrations of stimuli presented to the larvae overlaid with video frames of resultant behavioral responses. For looming stimuli, video frames from stimulus onset: 1, +1 s; 2, +1.8 s; 3, +2.4 s; and 4, +2.7 s. For directional drifting gratings: 1, +1.3 s; 2, +2.7 s; and 3, +4 s. For solenoid-induced mechano-acoustic tap stimuli: 1, +0.1 s and 2, +0.17 s. For *Paramecia*: red circles indicate tracked *Paramecia*. Scale bar, 5 mm.

(C) Schematic illustrating extraction of kinematic variables and hunting performance metrics. Distance and orientation gains are computed from changes between start and end of each swim bout in a hunting routine. For details, see STAR Methods.

(D–D") Video frames from a hunting routine that ended with a successful capture. The target *Paramecium* is indicated with a yellow label. Scale bar, 1 mm.

(E–E") A hunting routine that was aborted. Frame timestamps are relative to time of hunting onset (convergent saccade).

(F) Behavioral kinematics for routine in (D). Black asterisk marks the convergent saccade at the beginning of the hunting routine. Lower plot shows gain values for each swim bout. In this case, distance and orientation gains are positive for all bouts.

(G) Data for the aborted hunting routine in (E). The final bout has negative distance and orientation gain, and vergence concurrently returns to baseline levels.

pursuit following a negative-gain bout and ocular vergence decreased back to baseline levels within 0.35 ± 0.01 s following such bouts (Figure S4K), indicating that they predominantly occur at the termination of hunting epochs. Notably, hunting routines could be aborted at a variety of stages as measured by the number of bouts performed (Figure S4J) or remaining distance to prey (Figure S4O').

We also recorded other behaviors that would enable us to evaluate the specificity of ablation phenotypes (Figure 3B). Looming spots were presented in a fixed egocentric reference frame to evoke escape swims [37], whole-field drifting gratings evoked the optomotor response (OMR) [38], and tap stimuli were delivered to evoke vibrational-acoustic startle responses [39, 40].

NI Is Required to Sustain Hunting Sequences

To test the role of NI in hunting, we used a pulsed infrared laser to bilaterally ablate cells in the Ch-A domain, where type I neurons are localized, while attempting to minimize damage to the Ch-B region. Post hoc imaging was used to record the auto-fluorescent ablation "scar" (Figures S3A and S3B), and registration to the brain atlas allowed us to estimate the location of the lesion (Figures S3C and S3F). This revealed that the Ch-A domain was accurately targeted with minimal off-target damage. NI-ablated larvae (n = 8) and control fish (which underwent the same procedure with the exception of laser ablation; n = 7) were tested for behavior 1 day after the procedure, at 7 dpf. In addition, we performed "sham" ablations, medially adjacent to NI, as a control for off-target, laser-mediated damage in the hindbrain (Figures S3D, S3E, and S3G; n = 4).

Analysis of prey consumption revealed that NI-ablated larvae showed reduced hunting performance (Figures 4A and 4B). By contrast, sham ablations produced no prey-catching deficit (Figure S3H). To explore the basis for this, we next assessed whether ablated fish displayed defects in prey perception or reduced motivation to feed. However, the rate of initiation of hunting routines appeared unchanged as a result of NI ablation (Figures 4D and S4I). Likewise, the spatial location of target prey at the onset of hunting and at the time of initiation of capture swims did not differ between groups (Figure S4N). These results indicate that NI-ablated larvae show normal rates of hunting initiation directed at prey in the normal range of distances and eccentricities but nonetheless capture fewer prey than control fish.

To uncover why NI-ablated larvae capture fewer prey, we used high-speed tracking data to analyze the progression of individual hunting sequences. This analysis identified a remarkably specific defect. By quantifying the probabilities of outcomes at each stage of the routines, we discovered that NI-ablated fish displayed a substantial increase in the likelihood of aborting their pursuit of prey (controls: 0.60 ± 0.04; ablated: 0.80 ± 0.03; p = 0.006; Figures 4F, 4G, and 4I). Notably, we did not observe differences in other aspects of hunting: when larvae did attempt a final capture maneuver, the selection of capture type (ram versus suction) and capture success rates were equivalent to controls (Figures 4H–4M). Based on this finding, we asked whether the increased propensity to abandon hunting routines might be related to poorer performance during prey tracking by evaluating distance gain and orientation gain. However, we observed no differences between groups (Figures S4L and S4M), indicating that NI-ablated fish can correctly localize their prey and execute precise goal-directed tracking movements during prey pursuit.

Ablated fish explored the arena with similar swim speeds and spatial distributions as compared to controls (Figures S4A–S4C), and kinematic features of individual swim bouts were unaffected (Figures S4P–S4T). Moreover, drifting gratings and "tap" stimuli effectively induced OMR and startle responses, with performance equivalent to controls (Figures S4D–S4H).

Studies in birds have shown that the cholinergic Ipc subnucleus of NI modulates contrast-response gain and threshold of tectal units to looming stimuli [41]. We therefore investigated whether NI modulates looming-evoked escape behavior in larval zebrafish by presenting control (n = 6) and NI-ablated (n = 6) larvae with looming spots at five contrast levels. Escape probability increased monotonically as a function of stimulus contrast, and NI-ablated larvae showed a significant reduction in contrast sensitivity (quantified by the *threshold* of psychometric logistic function fits; Figures S4U and S4V). The critical angular spot size triggering escapes and escape directionality were contrast invariant and were unaffected by NI ablation (Figures S4Y and S4Z).

In summary, ablation of NI caused specific deficits in hunting behavior and escape from looming threats. In the context of hunting, NI is specifically required for zebrafish to sustain prey tracking. NI does not appear to be required for prey detection or localization, hunting initiation, or sensorimotor performance during tracking or capture. In NI-ablated larvae, hunting is initiated normally, but there is a substantially elevated probability that they will abort the hunting sequence during prey tracking.

NI Responds to Looming and Prey-like Visual Stimuli

Our behavioral analyses revealed that NI is required for specific aspects of two tectally dependent, visually guided behaviors. Next, we sought to characterize NI neuronal activity in response to relevant visual cues. To do this, we used 2-photon functional calcium imaging with transgenic Tg(*elavl3*:H2B-GCaMP6s) larvae (Figure 5A). To examine how isthmic activity relates to that in its target regions, we imaged a field of view that included the isthmus, OT, and pretectal region encompassing AF7, on both sides of the brain (Figure 5B").

First, we examined responses to looming stimuli. Larvae (n = 3) were presented with dark looming spots as well as control stimuli that produced the same temporal profile of whole-field dimming but were of fixed size (subtending the same final angle at the eye as the looming spots; STAR Methods). This enabled identification of neurons that specifically respond to radially expanding looming spots, which evoke escapes, as opposed to non-specific dimming stimuli, which do not [37, 42]. To identify consistent visual response properties, we first built visual response vectors (VRVs) for single neurons by concatenating their mean fluorescence time series for each type of visual cue. Next, we used a two-stage clustering procedure to identify groups of cells with similar visual tuning (STAR Methods) [13]. Inspection of clusters revealed that they were homogeneous with respect to VRVs of constituent cells (Figure S5A).

We identified two clusters that responded to looming spots but showed minimal response to the control dimming stimulus (Figure S5A). These clusters differed in temporal dynamics, having either phasic ("loom") or sustained ("loom-long") loom-evoked activity. When we examined the anatomical distribution

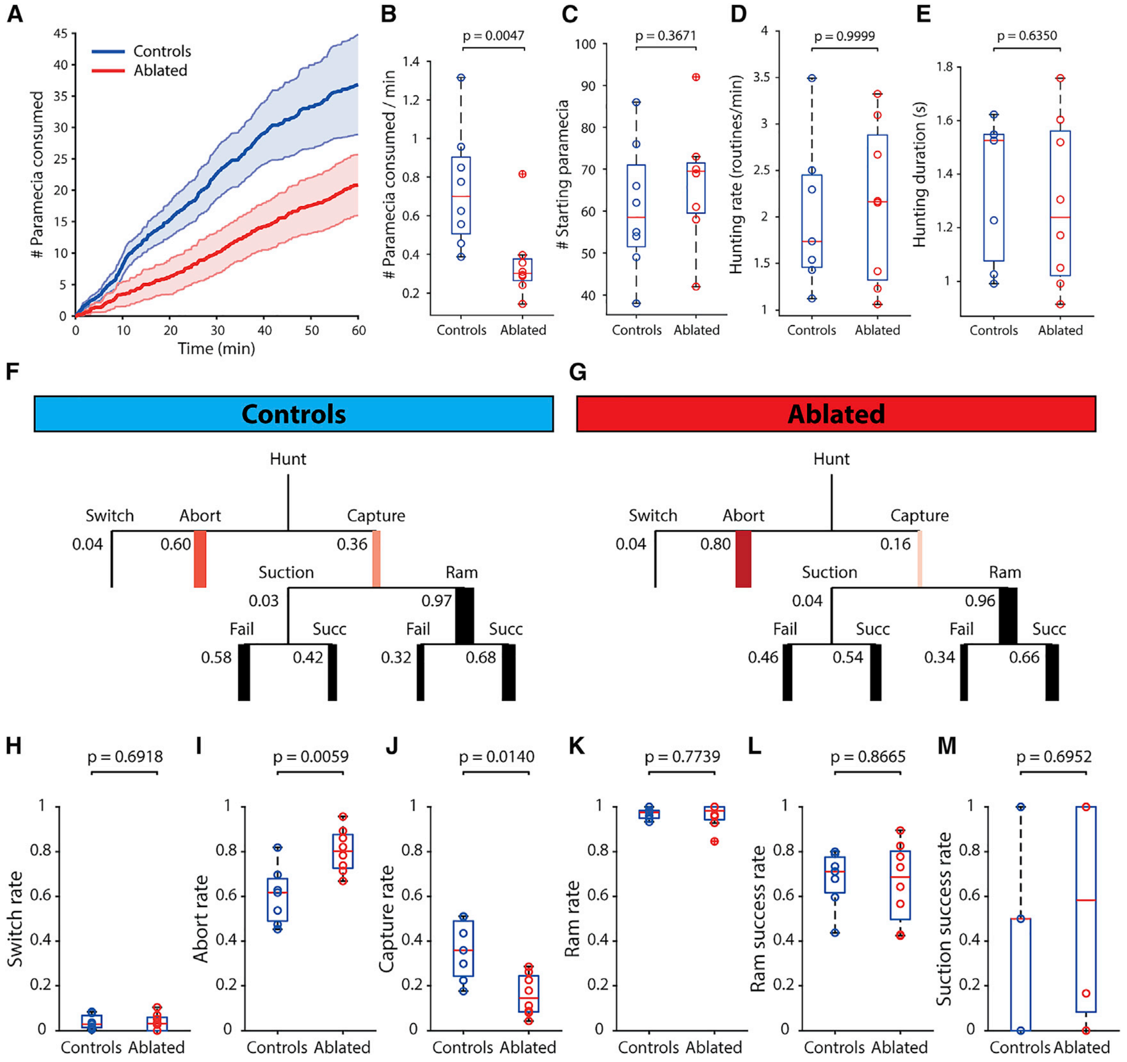

Figure 4. NI Is Required to Sustain Hunting Behavior

(A) Cumulative number of *Paramecia* consumed by NI-ablated and control larvae (mean ± SEM).

(B) Mean *Paramecia* consumption rates for duration of the experiment.

(C) Number of *Paramecia* at the start of each experiment.

(D) Rate of initiation of hunting routines.

(E) Hunting epoch durations, based on periods of elevated ocular vergence.

(F and G) Ethograms indicating progression of hunting routines for control (F) and NI-ablated (G) larvae. Branch thickness corresponds to mean probability for the corresponding event, which is also displayed numerically.

(H–M) Probabilities of events within hunting routines for control and ablated animals. Data points represent median values for each fish. Wilcoxon rank sum tests are shown. In NI-ablated larvae, there is a substantial increase in the probability of aborting prey tracking. Note that p values are shown without correction for multiple comparisons. However, elevated abort rate remains statistically significant, at $\alpha = 0.05$, with Bonferroni correction for multiple (6) comparisons.

See also Figures S3 and S4.

of these neurons, we found a large number of looming-responsive cells of both types in OT, in agreement with previous findings [37], as well as in pretectum (Figure S5B). Although only a small proportion of NI neurons were identified as visually responsive (30%) or assigned to visual clusters (7%; n = 926), looming-responsive neurons of both types were found within NI and were rather evenly distributed in both Ch-A and Ch-B domains (Figures S5C and S5D).

Next, we tested responses to prey-like visual cues by combining 2-photon imaging with a virtual hunting assay (Figures 5A and 5B) [13]. Briefly, Tg(*elavl3*:H2B-GCaMP6s) larvae (n = 9) were restrained in agarose gel but with their eyes and tail free to move, and prey-like cues were back projected onto a screen covering the frontal portion of visual space to evoke hunting responses. Stimuli were presented in closed loop such that the angular location of the small moving spot was updated in real time in response to the animal's behavior, which was tracked online using infrared cameras. We also presented light or dark whole-field flashes to detect luminance-responsive neurons.

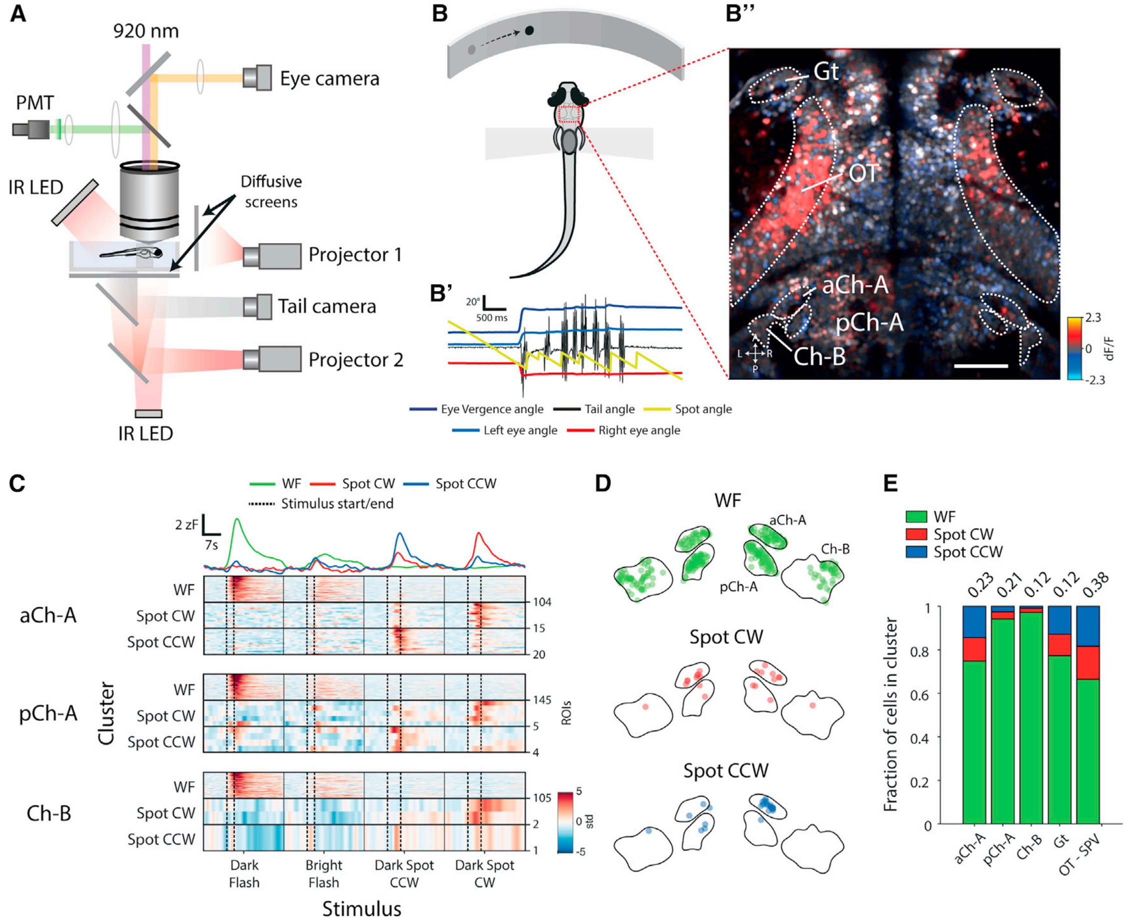

Figure 5. NI Neurons Respond to Looming and Prey-like Visual Stimuli

(A) Schematic of the 2-photon setup to record neuronal activity and visually evoked behavior in tethered larval zebrafish.

(B) Schematic of the virtual hunting assay. (B') Tail, eye, and stimulus dynamics during a tethered hunting routine are shown. The prey-like spot is moved to the midline when the fish converges its eyes and after subsequent swim bouts. (B'') Map of neuronal activity (ΔF/F) in response to a leftward moving prey-like spot is shown. Scale bar, 50 μm.

(C) Visual responses of isthmic neurons. Rasters show VRVs of cells assigned to visual clusters in the virtual hunting experiment. Top traces represent the mean VRV for all clustered isthmic neurons.

(D) Anatomical distribution of visually responsive cells in the isthmus, colored by cluster type. For illustration purposes, the distances between regions have been offset, but distances within each region are correct. Boundaries are drawn at a single dorsoventral level.

(E) Visual response types by brain region. Numbers on top show the fraction of cells in the brain region that were assigned cluster identities (aCh-A, n = 139; pCh-A, n = 154; Ch-B, n = 108; Gt, n = 194; OT-SPV, n = 3,703).

See also Figure S5.

Visually responsive neurons fell into three major clusters (Figure S5E). The largest was responsive to whole-field flash stimuli, "WF," and two clusters showed direction-selection responses to prey-like moving spots: "spot CW" and "spot CCW" (tuned to clockwise and counterclockwise motion, respectively). WF cells represented the majority of clustered neurons (84% versus 7% spot CW and 9% spot CCW; n = 31,980) and were widespread across the brain (Figure S5F). Spot-responsive neurons were also widespread, but their distribution showed marked lateralization, particularly in OT, where spot CW and spot CCW neurons were more abundant in the right and left hemispheres, respectively (Figure S5F).

Within NI, around half the cells were classified as visually responsive (45%; n = 2,258), and a smaller fraction were assigned to visual clusters (18%), in comparison to OT (78% visually responsive; 38% cluster-assigned; n = 9,778; Figure 5E). Of the NI cells that were assigned a visual cluster identity, the majority were of the WF type. However, a relatively large proportion of aCh-A neurons were responsive to prey-like visual stimuli (25%; versus 6% and 3% for pCh-A and Ch-B, respectively; Figures 5C–5E). By comparing the response time courses of aCh-A neurons with cells in OT, we deduced that these NI cells respond to prey-like cues in relatively rostral regions of the contralateral visual hemifield (Figure S5G). This is compatible with NI neurons receiving visual input from the ipsilateral OT, especially from its anterior domain.

Taken together, these results indicate that NI contains neurons responsive to both looming spots and prey-like stimuli. The former

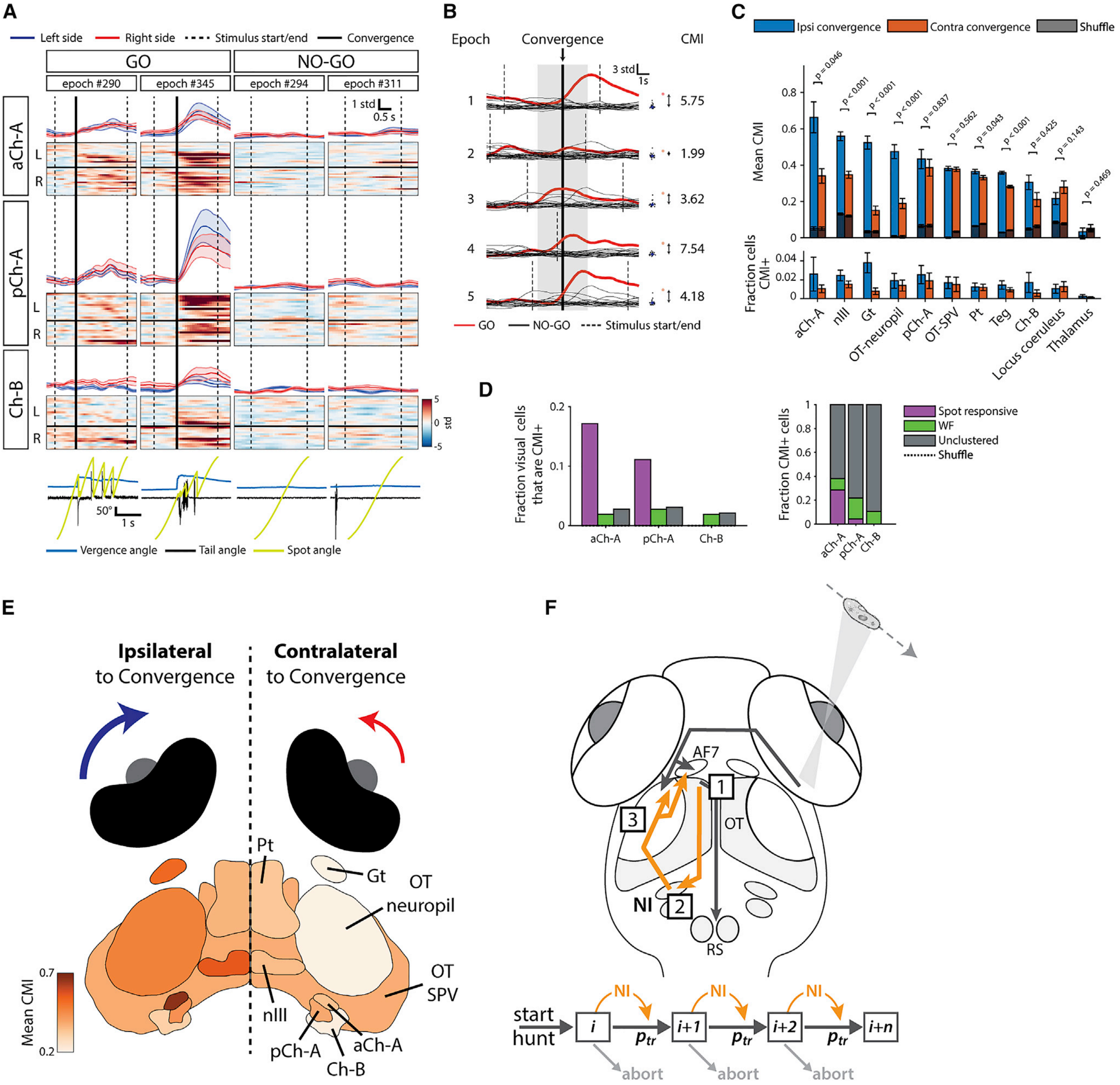

Figure 6. NI Neurons Are Recruited during Hunting Behavior

(A) Examples of NI activity in GO and NO-GO trials. Rasters show activity of all imaged neurons in the corresponding isthmic areas. Dashed lines mark stimulus presentation period, and thick black lines mark time of convergent saccade in GO trials. Upper traces show mean ± SEM for left and right cells. Bottom traces show tail and eye kinematics and spot location.

(B) Convergence modulation index (CMI) calculation, illustrated for an example neuron. Mean calcium fluorescence during a time window surrounding each convergence event (gray shading) is compared to values at the same time and for the same visual stimulus during NO-GO epochs. The distance between the GO and NO-GO values is measured in SDs of the NO-GO distribution. The CMI for the cell is then calculated as the mean of these distance metrics across GO epochs.

(C) Mean CMI and fraction of CMI+ cells (CMI > 3) for several imaged brain regions. Error bars show mean ± SEM. Gray bars indicate values expected by chance from shuffle analysis (see STAR Methods). Wilcoxon signed rank tests between each region's ipsilateral and contralateral convergence distributions.

(D) Left panel shows proportions of visually responsive isthmic neurons that are CMI+. Right panel shows the proportion of CMI+ cells in isthmus belonging to visual clusters.

(E) Brain areas colored by mean CMI for ipsilateral or contralateral convergences, as per values in (C).

(legend continued on next page)

are distributed across isthmic regions, similar to the distribution of type II cells, whereas prey-responsive neurons preferentially localize to the aCh-A domain, where type I cells are found.

NI Neurons Are Activated during Hunting

Because our ablation experiments demonstrated that NI is required to sustain hunting sequences, we next explored the hypothesis that NI neurons might show activity associated with the generation of hunting behavior.

To examine motor-related activity, we compared GO trials, where larvae responded to prey-like cues by initiating hunting (defined by eye convergence) [9, 13], to NO-GO trials where no response occurred (GO: 5.8% ± 3.6%; n = 9 fish). Alignment of GCaMP fluorescence to the onset of eye convergence in GO trials revealed that a proportion of NI neurons became activated at, or soon after, hunting initiation but displayed little activity during presentation of the same visual stimulus in NO-GO trials (Figures 6A and S6A).

As a principled means to distinguish convergence (motor) versus visual (sensory) activity, we computed a metric that compares calcium responses of individual cells in GO and NO-GO trials (convergence modulation index ["CMI"]; Figure 6B; STAR Methods). Briefly, for each GO trial, activity in a time window (±2 s) surrounding the convergent saccade was compared to activity at the same time, and for the same visual stimulus, in corresponding NO-GO trials. The mean of these difference measures across all GO trials represents the CMI score for the cell and quantifies the degree to which its activity is modulated by hunting behavior. "Sp-CMI" scores were computed in a similar way for spontaneous convergent saccades (n = 586; STAR Methods). Finally, because convergent saccades are frequently lateralized (the eye contralateral to target location shows a greater nasal rotation) [10, 13], we computed CMI separately for left- and right-eye-dominant convergences.

Comparing CMI scores across brain regions revealed that the aCh-A region of NI had the highest average score (Figures 6C and 6E). Other regions with high CMI included the oculomotor nucleus, which contains the medial rectus motoneurons directly responsible for nasal eye rotations. In the isthmus, pCh-A and Ch-B domains showed lower CMI but which nonetheless exceeded the level estimated by chance (shuffle values; Figure 6C). Moreover, CMI showed marked lateralization: higher values were observed in the brain hemisphere ipsilateral to the eye displaying greater convergence (Figures 6C and 6E). In other words, if the left eye makes a greater nasal rotation (to direct the binocular visual field toward prey on the right), it is the left aCh-A, oculomotor, and OT that show the strongest modulation in neural activity. Similar results were obtained when we examined mean Sp-CMI and the fraction of neurons in each region with high CMI and Sp-CMI scores (greater than 3, "CMI+"/"Sp-CMI+"; Figures 6C and S6B). Examination of single-trial responses showed that convergence-modulated neurons in aCh-A showed elevated activity in multiple GO trials, with a consistently lower level of activity in NO-GO epochs (Figure S6C). These results show that NI neurons, especially in the aCh-A domain, display motor-related neural activity and are recruited during hunting behavior.

Of the visually responsive isthmic neurons, those that responded to prey-like cues were more likely to show convergence-modulated activity. Specifically, a higher fraction of prey-responsive cells in the aCh-A (17%) and pCh-A (11%) domains were convergence-modulated (CMI+) as compared to WF or unclustered cells (2%–3%; Figure 6D). Reciprocally, although most CMI+ cells were not assigned to visual clusters, those that were were most likely to be prey responsive (Figure 6D, right).

Overall, these results indicate that NI neurons, principally in the aCh-A region where type I cells are found, are recruited at the onset of hunting behavior. Although visual and motor responses are largely segregated in NI, some neurons carry both sensory and motor signals related to hunting. We propose that NI recruitment during hunting modulates activity of tectal and pretectal neurons to promote the maintenance of prey-catching sequences (Figure 6F).

DISCUSSION

Anatomy and Connectivity of NI in Larval Zebrafish

We used gene expression analysis, single-cell labeling, and tract tracing to characterize the isthmic region of rhombomere 1 in larval zebrafish at 6 dpf. We identified two largely non-overlapping isthmic domains—Ch-A and Ch-B—defined by expression of cholinergic genes from either the CGLa or CGLb genomic locus, respectively [17]. The posterior-dorsal region of the Ch-B domain coexpresses Calretinin and likely corresponds to at least part of the larval SGN/V (likely homologous to mammalian parabrachial nucleus). Single neurons in this region made conspicuous projections to the ipsilateral lateral hypothalamus, in agreement with SGN/V projections described in adult zebrafish [19, 24, 43].

Our gene expression and connectivity data indicate that the cholinergic NI of larval zebrafish is located in the Ch-A domain of rhombomere 1. In addition to cholinergic markers, the anterior Ch-A region expresses *reelin*, a marker for NI in zebrafish [21, 22]. Crucially, our single-cell labeling, as well as retrograde and anterograde tracing experiments, revealed reciprocal projections between this isthmic region and the ipsilateral tectum, a defining feature of NI connectivity across vertebrates [12, 15]. Because some tectally projecting type II cells are also found in the rostral Ch-B region, we suggest this also forms part of NI.

Two Types of NI Projection Neuron

Using focal electroporation, we identified two distinct types of isthmic projection neuron. Type I cells are found in the anterior Ch-A region and make ascending ipsilateral projections in which

(F) Model for NI-mediated feedback facilitation acting to sustain hunting sequences. (1) Hunting behavior is initiated by tectal and/or pretectal neurons in response to prey-like visual cues. (2) Type I cells in ipsilateral NI are recruited by tecto-isthmic projections at hunting onset. (3) Type I cells project back to rostral OT and AF7 and potentiate neural activity, lowering the threshold for successive visuomotor outputs (goal-directed, prey-tracking maneuvers). Lower schematic illustrates how NI might function in a hunting-state-dependent manner to increase transition probability (p_{tr}) between successive visuomotor elements (boxes) within a behavioral sequence.
See also Figure S6.

single axons innervate both rostral OT and the pretectal AF7 neuropil. AF7 is a retinal arborization field that is likely presynaptic to the parvocellular superficial pretectal nucleus (PSp). NI has been shown to project to the pretectum in several teleost species [29, 44–50], including innervation of PSp in adult zebrafish [51]. Therefore, NI type I cells appear to represent a pattern of isthmo-pretectal connectivity that is conserved at least across teleosts. However, it has also been suggested that PSp is equivalent to the mammalian lateral geniculate nucleus (LGN), and PBg axons innervate LGN (as well as superior colliculus) in mammals [52]. To our knowledge, this is the first study to demonstrate that a single type of NI neuron innervates both pretectum and OT.

Type II cells project bilaterally to OT, establishing larger terminal arbors over a broader region of the tectal retinotopic map and innervating deeper laminae than type I cells. Although NI and PBg projections to contralateral as well as ipsilateral OT and SC have been described in several species [14], in most cases, it is unknown whether single NI cells innervate both tectal lobes. One exception is a study that found that the majority of NI cells in tongue-projecting salamanders project bilaterally to OT [53]. The targeting of deeper laminae by type II cells suggests that they may function to modulate tectal response properties or gate tectal premotor outputs. Notably, type II somata were found within both cholinergic expression domains. Although the biochemical properties of zebrafish Chata and Chatb isoforms have not been studied, it is possible this corresponds to functional diversity within type II neurons.

NI Modulates Tectally Mediated Goal-Directed Behaviors

Across vertebrates, OT and SC controls orienting and avoidance responses [54, 55] and tectal involvement in both prey-catching and loom-avoidance has been established in zebrafish [7, 13, 37, 42]. We demonstrate that NI is involved in specific and distinct features of both hunting and loom-avoidance.

Zebrafish robustly respond to looming spots—which likely represent approaching predators or objects on a collision course—with directional escapes [37], a behavior that is conserved across species. We show that escape probability, but not critical angular size of the looming disc, is modulated by stimulus contrast and that NI is required for normal contrast sensitivity. This behavioral result complements physiological studies in teleosts, showing loom-evoked activity in NI [56] and birds, showing that the cholinergic Ipc NI subnucleus modulates gain of tectal looming detectors [41].

In the context of prey-catching behavior, we find that NI is specifically required for larval zebrafish to sustain target pursuit during hunting sequences. A role for NI in prey-catching has been suggested by various studies, especially in amphibians. It has been reported that NI ablation causes visual scotoma, leading to loss of behavioral responses to prey-like or looming stimuli in a portion of visual space [57, 58], although this result has been contested [59]. Although NI has been shown to relay visual information between the tectal lobes to mediate binocular visual responses in rostral tectum [60], NI does not seem to be required for depth perception in hunting frogs or toads [59]. In addition, studies in the salamander and cat have suggested a role for NI and PBg in tracking moving visual targets [61, 62]. Interestingly, we see no evidence for defects in sensory processing of prey following NI ablation. Ablated larvae initiated hunting routines at the same rate as controls and directed toward prey within the same reactive perceptive field, showing no signs of scotoma. Distance and orientation gains were unchanged, indicating normal ability to both spatially localize and track prey. Finally, ablated larvae fixated prey within the same strike zone as controls and produced equally accurate capture swims, arguing against defects in binocular localization or depth perception [9].

NI Sustains Hunting Behavior

By analyzing predator-prey interactions and the evolution of individual hunting sequences, we found that NI is required for larvae to sustain hunting routines. When NI is ablated, zebrafish are substantially more likely to abort hunting routines prematurely during target pursuit. Studies in many species have identified brain regions, cell types or circuits involved in driving behavior, or modulating aspects such as response probability, latency, or action selection (e.g., [63–66]). However, very little is known about how animals sustain ongoing behavioral routines that require a sequence of successive sensorimotor transformations. Our data indicate that NI functions to sustain hunting state, such that the probability of successive prey-tracking responses is increased. The specificity of this behavioral phenotype, and absence of changes in the commencement of hunting routines, indicates that the motivation to feed and initiation of prey-catching are operationally distinct to the maintenance of hunting state once a routine is underway. Why a hunting zebrafish should sometimes abandon pursuit of its prey is not clear. From our data, we could not predict aborted routines from either sensory features (including location, velocity, or local density of prey) or tracking performance (distance and orientation gain of bouts prior to abort; data not shown). A simple model is that the execution of each element in a hunting sequence is stochastic. In this scheme, NI increases the transition probability of progressing to the next element (tracking swim) in the sequence, with a concomitant decrease in the probability of aborting the routine (Figure 6F). Below, we discuss a circuit mechanism by which NI could serve this function.

Sensory and Motor Response Properties of NI Neurons

Although we could not specifically target type I versus type II cells for laser ablation, our data suggest distinct functional roles for these isthmic projection neurons. Across species, NI and PBg appears to respond to various visual stimuli, including luminance changes [61, 67], moving targets [62], and looming objects [56, 68, 69]. In agreement with this, we find that NI neurons in larval zebrafish respond to whole-field luminance changes, and a smaller subset responds to prey-like moving spots or looming stimuli. Looming-responsive cells showed an anatomical distribution most similar to type II neurons. In future studies, it will be interesting to examine whether type II afferents modulate tectal looming detectors and/or influence visuomotor "gating" and tectofugal transmission to downstream tegmental circuits to trigger looming-evoked escapes.

Neural activity in the aCh-A domain, as well as the projection patterns of type I neurons, strongly support a role for this NI cell type in modulation of hunting. The majority of spot-responsive NI neurons localized to aCh-A and the timing of their activity

indicates that these cells respond to visual targets in rostral regions of the contralateral visual hemifield. This is compatible with type I cells receiving afferent input preferentially from rostral OT, as indicated by the tecto-isthmic circuitry we identified using PA-GFP-assisted circuit tracing. Moreover, because type I cells elaborate terminal arbors in anterior OT, this supports the existence of a "homotopic" feedback circuit with rostral tectum, similar to the "point-to-point" feedback connectivity between OT and NI described in other species [28]. By using a naturalistic hunting assay for tethered larvae, our study is one of few to have recorded NI activity during behavior. By comparing activity in response (GO) versus non-response (NO-GO) trials, a substantial motor-related signal was detected in aCh-A, associated with the generation of hunting behavior. The ipsilateral tecto-isthmic projection, terminating at the location of type I dendrites, may mediate recruitment of type I cells at hunting onset. We propose that the requirement for NI for progression of hunting sequences is mediated by this specific type of isthmic neuron.

Model for Maintenance of Hunting State

How does NI function to promote the progression of hunting routines? We suggest that, following initiation of hunting, NI provides state-dependent feedback to anterior OT and pretectum, which potentiates visually evoked neuronal activity and thereby increases the probability of successive behavioral responses to target prey (Figure 6F).

A number of our findings are compatible with this model. (1) NI ablation revealed a specific requirement to sustain hunting routines but no defects in prey detection and localization or in hunting initiation. (2) NI shows elevated activity associated with hunting behavior. Ablation data suggest this activity is not required for hunting initiation but rather to maintain prey-tracking sequences. (3) This motor signal is strongest in the aCh-A domain where type I cells are located. (4) Anatomy and physiology data support the existence of a homotopic, ipsilateral feedback circuit between rostral OT and/or pretectum, where prey are represented, and type I cells in NI. (5) Isthmic gene expression indicates NI cells might co-release glutamate and acetylcholine, compatible with an excitatory effect at target loci.

In terms of synaptic transmission, studies in fish [70], amphibians [71, 72], and mammals [73] indicate that acetylcholine release from NI and PBg axon terminals acts presynaptically on RGC afferent terminals in OT and SC to facilitate glutamate release and thereby enhance retinotectal transmission. Type I axons targeted the superficial retinorecipient layers of the ipsilateral OT. Therefore, following NI recruitment, such a mechanism could mediate feedback facilitation of visually evoked activity in OT and/or pretectum in a hunting-state-dependent manner. By amplifying responses to prey, NI would function to increase the probability that tectal efferent circuits reach threshold to produce successive visuomotor (orienting) responses. In this way, NI would operationally act to promote the continuation of prey tracking.

The compact terminal arbors of type I neurons suggest that modulation of tectal activity is likely to be spatially specific. Isthmo-tectal circuitry is thought to comprise a midbrain network controlling spatial attention and has been well studied in birds [74]. Here, cholinergic NI neurons in Ipc and SLu provide spatially precise focal amplification of activity at a specific point within the tectal space map. Meanwhile, broad GABAergic projections from the Imc subnucleus of NI to OT are thought to implement global competitive inhibition that suppresses responses to competitor stimuli elsewhere in the visual field [75]. This circuitry implements a "winner-takes-all" mechanism to direct attention to the highest priority location in the visual scene. Although we did not identify a distinct GABAergic NI subnucleus in rhombomere 1, it is possible that such a nucleus is located more rostrally, for example, in the mesencephalic tegmentum. Nonetheless, because cholinergic type I cells establish compact and likely topographically mapped terminal arbors, it is probable that they mediate spatially specific response enhancement in larval zebrafish OT. It is currently unknown whether larval zebrafish display spatial attention. If NI were required for such a function, this might predict an increased probability of aborting hunting routines under conditions of high prey density (more competitor stimuli) in ablated animals. Although we did not observe this, it may be that prey density was not sufficiently high in our assay. Taken together, our results are compatible with a model in which NI functions to sustain hunting behavior by providing state-dependent feedback facilitation to OT and/or pretectum that effectively reduces the threshold for subsequent orienting responses within the visuomotor chain. In subsequent studies, it will be interesting to directly examine the proposed spatially specific feedback facilitation, as well as the relative contributions of isthmo-tectal versus isthmo-pretectal circuits.

STAR★METHODS

Detailed methods are provided in the online version of this paper and include the following:

- KEY RESOURCES TABLE
- CONTACT FOR REAGENT AND RESOURCE SHARING
- EXPERIMENTAL MODEL AND SUBJECT DETAILS
- METHOD DETAILS
  - Whole-mount in situ hybridization and immunohistochemistry
  - Lipophilic dye tracing
  - Focal electroporation and neuronal tracing
  - 3D image registration
  - 2-photon calcium imaging combined with behavioral tracking
  - Calcium imaging analysis
  - Photoactivation of PA-GFP
  - Laser ablations
  - Monitoring free-swimming behavior
  - Free-swim behavior analysis
- QUANTIFICATION AND STATISTICAL ANALYSIS
- DATA AND SOFTWARE AVAILABILITY

SUPPLEMENTAL INFORMATION

Supplemental Information can be found online at https://doi.org/10.1016/j.cub.2019.04.064.

ACKNOWLEDGMENTS

The authors thank members of our lab, David Attwell, Steve Wilson, and Tiago Branco for helpful discussions and critical feedback on the manuscript and UCL Fish Facility staff for fish care and husbandry. Funding was provided

from a Sir Henry Dale Fellowship from the Royal Society & Wellcome Trust (101195/Z/13/Z) to I.H.B., a UCL Excellence Fellowship to I.H.B., and a UCL IMPACT studentship to P.H.

AUTHOR CONTRIBUTIONS

Conceptualization and Methodology, P.M.H. and I.H.B.; Investigation, P.M.H., S.E.J., and N.R.; Analysis, P.M.H.; Writing, P.M.H. and I.H.B.; Supervision and Funding Acquisition, I.H.B.

DECLARATION OF INTERESTS

The authors declare no competing interests.

Received: December 19, 2018
Revised: March 4, 2019
Accepted: April 25, 2019
Published: May 16, 2019

REFERENCES

1. Berman, G.J. (2018). Measuring behavior across scales. BMC Biol. *16*, 23.
2. Long, M.A., Jin, D.Z., and Fee, M.S. (2010). Support for a synaptic chain model of neuronal sequence generation. Nature *468*, 394–399.
3. Seeds, A.M., Ravbar, P., Chung, P., Hampel, S., Midgley, F.M., Jr., Mensh, B.D., and Simpson, J.H. (2014). A suppression hierarchy among competing motor programs drives sequential grooming in Drosophila. eLife *3*, e02951.
4. Evans, H.E. (1966). The behavior patterns of solitary wasps. Annu. Rev. Entomol. *11*, 123–154.
5. Ewert, J.P. (1974). The neural basis of visually guided behavior. Sci. Am. *230*, 34–42.
6. Budick, S.A., and O'Malley, D.M. (2000). Locomotor repertoire of the larval zebrafish: swimming, turning and prey capture. J. Exp. Biol. *203*, 2565–2579.
7. Gahtan, E., Tanger, P., and Baier, H. (2005). Visual prey capture in larval zebrafish is controlled by identified reticulospinal neurons downstream of the tectum. J. Neurosci. *25*, 9294–9303.
8. Borla, M.A., Palecek, B., Budick, S., and O'Malley, D.M. (2002). Prey capture by larval zebrafish: evidence for fine axial motor control. Brain Behav. Evol. *60*, 207–229.
9. Bianco, I.H., Kampff, A.R., and Engert, F. (2011). Prey capture behavior evoked by simple visual stimuli in larval zebrafish. Front. Syst. Neurosci. *5*, 101.
10. Trivedi, C.A., and Bollmann, J.H. (2013). Visually driven chaining of elementary swim patterns into a goal-directed motor sequence: a virtual reality study of zebrafish prey capture. Front. Neural Circuits *7*, 86.
11. Marques, J.C., Lackner, S., Félix, R., and Orger, M.B. (2018). Structure of the zebrafish locomotor repertoire revealed with unsupervised behavioral clustering. Curr. Biol. *28*, 181–195.e5.
12. Butler, A.B., and Hodos, W. (2005). Comparative Vertebrate Neuroanatomy: Evolution and Adaptation, Second Edition (Hoboken, N.J: Wiley-Interscience).
13. Bianco, I.H., and Engert, F. (2015). Visuomotor transformations underlying hunting behavior in zebrafish. Curr. Biol. *25*, 831–846.
14. Wang, S.R. (2003). The nucleus isthmi and dual modulation of the receptive field of tectal neurons in non-mammals. Brain Res. Brain Res. Rev. *41*, 13–25.
15. Gruberg, E., Dudkin, E., Wang, Y., Marín, G., Salas, C., Sentis, E., Letelier, J., Mpodozis, J., Malpeli, J., Cui, H., et al. (2006). Influencing and interpreting visual input: the role of a visual feedback system. J. Neurosci. *26*, 10368–10371.
16. Wu, D., and Hersh, L.B. (1994). Choline acetyltransferase: celebrating its fiftieth year. J. Neurochem. *62*, 1653–1663.
17. Hong, E., Santhakumar, K., Akitake, C.A., Ahn, S.J., Thisse, C., Thisse, B., Wyart, C., Mangin, J.M., and Halpern, M.E. (2013). Cholinergic left-right asymmetry in the habenulo-interpeduncular pathway. Proc. Natl. Acad. Sci. USA *110*, 21171–21176.
18. Marquart, G.D., Tabor, K.M., Brown, M., Strykowski, J.L., Varshney, G.K., LaFave, M.C., Mueller, T., Burgess, S.M., Higashijima, S., and Burgess, H.A. (2015). A 3D searchable database of transgenic zebrafish Gal4 and Cre lines for functional neuroanatomy studies. Front. Neural Circuits *9*, 78.
19. Mueller, T., Vernier, P., and Wullimann, M.F. (2004). The adult central nervous cholinergic system of a neurogenetic model animal, the zebrafish Danio rerio. Brain Res. *1011*, 156–169.
20. Castro, A., Becerra, M., Manso, M.J., and Anadón, R. (2006). Calretinin immunoreactivity in the brain of the zebrafish, Danio rerio: distribution and comparison with some neuropeptides and neurotransmitter-synthesizing enzymes. II. Midbrain, hindbrain, and rostral spinal cord. J. Comp. Neurol. *494*, 792–814.
21. Costagli, A., Kapsimali, M., Wilson, S.W., and Mione, M. (2002). Conserved and divergent patterns of Reelin expression in the zebrafish central nervous system. J. Comp. Neurol. *450*, 73–93.
22. Volkmann, K., Chen, Y.Y., Harris, M.P., Wullimann, M.F., and Köster, R.W. (2010). The zebrafish cerebellar upper rhombic lip generates tegmental hindbrain nuclei by long-distance migration in an evolutionary conserved manner. J. Comp. Neurol. *518*, 2794–2817.
23. Zou, M., Friedrich, R.W., and Bianco, I.H. (2016). Targeted electroporation in embryonic, larval, and adult zebrafish. Methods Mol. Biol. *1451*, 259–269.
24. Yáñez, J., Souto, Y., Piñeiro, L., Folgueira, M., and Anadón, R. (2017). Gustatory and general visceral centers and their connections in the brain of adult zebrafish: a carbocyanine dye tract-tracing study. J. Comp. Neurol. *525*, 333–362.
25. Gruberg, E.R., and Udin, S.B. (1978). Topographic projections between the nucleus isthmi and the tectum of the frog Rana pipiens. J. Comp. Neurol. *179*, 487–500.
26. Wang, S.R., Yan, K., Wang, Y.T., Jiang, S.Y., and Wang, X.S. (1983). Neuroanatomy and electrophysiology of the lacertilian nucleus isthmi. Brain Res. *275*, 355–360.
27. Künzle, H., and Schnyder, H. (1984). The isthmus-tegmentum complex in the turtle and rat: a comparative analysis of its interconnections with the optic tectum. Exp. Brain Res. *56*, 509–522.
28. Wang, Y., Luksch, H., Brecha, N.C., and Karten, H.J. (2006). Columnar projections from the cholinergic nucleus isthmi to the optic tectum in chicks (Gallus gallus): a possible substrate for synchronizing tectal channels. J. Comp. Neurol. *494*, 7–35.
29. Sakamoto, N., Ito, H., and Ueda, S. (1981). Topographic projections between the nucleus isthmi and the optic tectum in a teleost. Navodon modestus. Brain Res. *224*, 225–234.
30. Bianco, I.H., Ma, L.H., Schoppik, D., Robson, D.N., Orger, M.B., Beck, J.C., Li, J.M., Schier, A.F., Engert, F., and Baker, R. (2012). The tangential nucleus controls a gravito-inertial vestibulo-ocular reflex. Curr. Biol. *22*, 1285–1295.
31. Patterson, B.W., Abraham, A.O., MacIver, M.A., and McLean, D.L. (2013). Visually guided gradation of prey capture movements in larval zebrafish. J. Exp. Biol. *216*, 3071–3083.
32. Niell, C.M., and Smith, S.J. (2005). Functional imaging reveals rapid development of visual response properties in the zebrafish tectum. Neuron *45*, 941–951.
33. Robles, E., Laurell, E., and Baier, H. (2014). The retinal projectome reveals brain-area-specific visual representations generated by ganglion cell diversity. Curr. Biol. *24*, 2085–2096.
34. Semmelhack, J.L., Donovan, J.C., Thiele, T.R., Kuehn, E., Laurell, E., and Baier, H. (2014). A dedicated visual pathway for prey detection in larval zebrafish. eLife *3*, e04878.
35. McElligott, M.B., and O'malley, D.M. (2005). Prey tracking by larval zebrafish: axial kinematics and visual control. Brain Behav. Evol. *66*, 177–196.

36. Westphal, R.E., and O'Malley, D.M. (2013). Fusion of locomotor maneuvers, and improving sensory capabilities, give rise to the flexible homing strikes of juvenile zebrafish. Front. Neural Circuits *7*, 108.

37. Dunn, T.W., Gebhardt, C., Naumann, E.A., Riegler, C., Ahrens, M.B., Engert, F., and Del Bene, F. (2016). Neural circuits underlying visually evoked escapes in larval zebrafish. Neuron *89*, 613–628.

38. Neuhauss, S.C., Biehlmaier, O., Seeliger, M.W., Das, T., Kohler, K., Harris, W.A., and Baier, H. (1999). Genetic disorders of vision revealed by a behavioral screen of 400 essential loci in zebrafish. J. Neurosci. *19*, 8603–8615.

39. Kimmel, C.B., Patterson, J., and Kimmel, R.O. (1974). The development and behavioral characteristics of the startle response in the zebra fish. Dev. Psychobiol. *7*, 47–60.

40. Burgess, H.A., and Granato, M. (2007). Sensorimotor gating in larval zebrafish. J. Neurosci. *27*, 4984–4994.

41. Asadollahi, A., and Knudsen, E.I. (2016). Spatially precise visual gain control mediated by a cholinergic circuit in the midbrain attention network. Nat. Commun. *7*, 13472.

42. Temizer, I., Donovan, J.C., Baier, H., and Semmelhack, J.L. (2015). A visual pathway for looming-evoked escape in larval zebrafish. Curr. Biol. *25*, 1823–1834.

43. Clemente, D., Porteros, A., Weruaga, E., Alonso, J.R., Arenzana, F.J., Aijón, J., and Arévalo, R. (2004). Cholinergic elements in the zebrafish central nervous system: histochemical and immunohistochemical analysis. J. Comp. Neurol. *474*, 75–107.

44. Ito, H., Sakamoto, N., and Takatsuji, K. (1982). Cytoarchitecture, fiber connections, and ultrastructure of nucleus isthmi in a teleost (Navodon modestus) with a special reference to degenerating isthmic afferents from optic tectum and nucleus pretectalis. J. Comp. Neurol. *205*, 299–311.

45. Murakami, T., Morita, Y., and Ito, H. (1986). Cytoarchitecture and fiber connections of the superficial pretectum in a teleost, Navodon modestus. Brain Res. *373*, 213–221.

46. Striedter, G.F., and Northcutt, R.G. (1989). Two distinct visual pathways through the superficial pretectum in a percomorph teleost. J. Comp. Neurol. *283*, 342–354.

47. King, W.M., and Schmidt, J.T. (1993). Nucleus isthmi in goldfish: in vitro recordings and fiber connections revealed by HRP injections. Vis. Neurosci. *10*, 419–437.

48. Pérez, S.E., Yáñez, J., Marín, O., Anadón, R., González, A., and Rodríguez-Moldes, I. (2000). Distribution of choline acetyltransferase (ChAT) immunoreactivity in the brain of the adult trout and tract-tracing observations on the connections of the nuclei of the isthmus. J. Comp. Neurol. *428*, 450–474.

49. Xue, H.G., Yamamoto, N., Yoshimoto, M., Yang, C.Y., and Ito, H. (2001). Fiber connections of the nucleus isthmi in the carp (Cyprinus carpio) and tilapia (Oreochromis niloticus). Brain Behav. Evol. *58*, 185–204.

50. Folgueira, M., Anadón, R., and Yáñez, J. (2008). The organization of the pretectal nuclei in the trout: a revision based on experimental holodological studies. Brain Res. Bull. *75*, 251–255.

51. Yáñez, J., Suárez, T., Quelle, A., Folgueira, M., and Anadón, R. (2018). Neural connections of the pretectum in zebrafish (Danio rerio). J. Comp. Neurol. *526*, 1017–1040.

52. Harting, J.K., Van Lieshout, D.P., Hashikawa, T., and Weber, J.T. (1991). The parabigeminogeniculate projection: connectional studies in eight mammals. J. Comp. Neurol. *305*, 559–581.

53. Wiggers, W. (1998). Isthmotectal connections in plethodontid salamanders. J. Comp. Neurol. *395*, 261–272.

54. Dean, P., Redgrave, P., and Westby, G.W. (1989). Event or emergency? Two response systems in the mammalian superior colliculus. Trends Neurosci. *12*, 137–147.

55. Northmore, D. (2011). In Optic tectum. Encyclopedia of Fish Physiology: From Genome to Environment, A. Farrell, ed. (Elsevier), pp. 131–142.

56. Gallagher, S.P., and Northmore, D.P.M. (2006). Responses of the teleostean nucleus isthmi to looming objects and other moving stimuli. Vis. Neurosci. *23*, 209–219.

57. Caine, H.S., and Gruberg, E.R. (1985). Ablation of nucleus isthmi leads to loss of specific visually elicited behaviors in the frog Rana pipiens. Neurosci. Lett. *54*, 307–312.

58. Gruberg, E.R., Wallace, M.T., Caine, H.S., and Mote, M.I. (1991). Behavioral and physiological consequences of unilateral ablation of the nucleus isthmi in the leopard frog. Brain Behav. Evol. *37*, 92–103.

59. Collett, T.S., Udin, S.B., and Finch, D.J. (1987). A possible mechanism for binocular depth judgements in anurans. Exp. Brain Res. *66*, 35–40.

60. Glasser, S., and Ingle, D. (1978). The nucleus isthmus as a relay station in the ipsilateral visual projection to the frog's optic tectum. Brain Res. *159*, 214–218.

61. Wiggers, W., and Roth, G. (1991). Anatomy, neurophysiology and functional aspects of the nucleus isthmi in salamanders of the family Plethodontidae. J. Comp. Physiol. A *169*, 165–176.

62. Cui, H., and Malpeli, J.G. (2003). Activity in the parabigeminal nucleus during eye movements directed at moving and stationary targets. J. Neurophysiol. *89*, 3128–3142.

63. Ewert, J.P., Buxbaum-Conradi, H., Dreisvogt, F., Glagow, M., Merkel-Harff, C., Röttgen, A., Schürg-Pfeiffer, E., and Schwippert, W.W. (2001). Neural modulation of visuomotor functions underlying prey-catching behaviour in anurans: perception, attention, motor performance, learning. Comp. Biochem. Physiol. A Mol. Integr. Physiol. *128*, 417–461.

64. Lovett-Barron, M., Andalman, A.S., Allen, W.E., Vesuna, S., Kauvar, I., Burns, V.M., and Deisseroth, K. (2017). Ancestral circuits for the coordinated modulation of brain state. Cell *171*, 1411–1423.e17.

65. Li, Y., Zeng, J., Zhang, J., Yue, C., Zhong, W., Liu, Z., Feng, Q., and Luo, M. (2018). Hypothalamic circuits for predation and evasion. Neuron *97*, 911–924.e5.

66. Kohl, J., Babayan, B.M., Rubinstein, N.D., Autry, A.E., Marin-Rodriguez, B., Kapoor, V., Miyamishi, K., Zweifel, L.S., Luo, L., Uchida, N., and Dulac, C. (2018). Functional circuit architecture underlying parental behaviour. Nature *556*, 326–331.

67. Northmore, D.P., and Gallagher, S.P. (2003). Functional relationship between nucleus isthmi and tectum in teleosts: synchrony but no topography. Vis. Neurosci. *20*, 335–348.

68. Mysore, S.P., and Knudsen, E.I. (2014). Descending control of neural bias and selectivity in a spatial attention network: rules and mechanisms. Neuron *84*, 214–226.

69. Asadollahi, A., Mysore, S.P., and Knudsen, E.I. (2010). Stimulus-driven competition in a cholinergic midbrain nucleus. Nat. Neurosci. *13*, 889–895.

70. King, W.M., and Schmidt, J.T. (1991). The long latency component of retinotectal transmission: enhancement by stimulation of nucleus isthmi or tectobulbar tract and block by nicotinic cholinergic antagonists. Neuroscience *40*, 701–712.

71. Titmus, M.J., Tsai, H.J., Lima, R., and Udin, S.B. (1999). Effects of choline and other nicotinic agonists on the tectum of juvenile and adult Xenopus frogs: a patch-clamp study. Neuroscience *91*, 753–769.

72. Dudkin, E.A., and Gruberg, E.R. (2003). Nucleus isthmi enhances calcium influx into optic nerve fiber terminals in Rana pipiens. Brain Res. *969*, 44–52.

73. Binns, K.E., and Salt, T.E. (2000). The functional influence of nicotinic cholinergic receptors on the visual responses of neurones in the superficial superior colliculus. Vis. Neurosci. *17*, 283–289.

74. Knudsen, E.I. (2018). Neural circuits that mediate selective attention: a comparative perspective. Trends Neurosci. *41*, 789–805.

75. Marín, G., Salas, C., Sentis, E., Rojas, X., Letelier, J.C., and Mpodozis, J. (2007). A cholinergic gating mechanism controlled by competitive interactions in the optic tectum of the pigeon. J. Neurosci. *27*, 8112–8121.

76. Vladimirov, N., Mu, Y., Kawashima, T., Bennett, D.V., Yang, C.T., Looger, L.L., Keller, P.J., Freeman, J., and Ahrens, M.B. (2014). Light-sheet functional imaging in fictively behaving zebrafish. Nat. Methods *11*, 883–884.

77. Wen, L., Wei, W., Gu, W., Huang, P., Ren, X., Zhang, Z., Zhu, Z., Lin, S., and Zhang, B. (2008). Visualization of monoaminergic neurons and neurotoxicity of MPTP in live transgenic zebrafish. Dev. Biol. *314*, 84–92.
78. Masai, I., Lele, Z., Yamaguchi, M., Komori, A., Nakata, A., Nishiwaki, Y., Wada, H., Tanaka, H., Nojima, Y., Hammerschmidt, M., et al. (2003). N-cadherin mediates retinal lamination, maintenance of forebrain compartments and patterning of retinal neurites. Development *130*, 2479–2494.
79. Mueller, T., and Guo, S. (2009). The distribution of GAD67-mRNA in the adult zebrafish (teleost) forebrain reveals a prosomeric pattern and suggests previously unidentified homologies to tetrapods. J. Comp. Neurol. *516*, 553–568.
80. Chong, S.W., Nguyen, T.T.H., Chu, L.T., Jiang, Y.J., and Korzh, V. (2005). Zebrafish id2 developmental expression pattern contains evolutionary conserved and species-specific characteristics. Dev. Dyn. *234*, 1055–1063.
81. Avants, B.B., Tustison, N.J., Song, G., Cook, P.A., Klein, A., and Gee, J.C. (2011). A reproducible evaluation of ANTs similarity metric performance in brain image registration. Neuroimage *54*, 2033–2044.
82. Longair, M.H., Baker, D.A., and Armstrong, J.D. (2011). Simple Neurite Tracer: open source software for reconstruction, visualization and analysis of neuronal processes. Bioinformatics *27*, 2453–2454.
83. Brainard, D.H. (1997). The Psychophysics Toolbox. Spat. Vis. *10*, 433–436.
84. Prins, N., and Kingdom, F.A.A. (2018). Applying the model-comparison approach to test specific research hypotheses in psychophysical research using the Palamedes toolbox. Front. Psychol. *9*, 1250.
85. Kawashima, T., Zwart, M.F., Yang, C.T., Mensh, B.D., and Ahrens, M.B. (2016). The serotonergic system tracks the outcomes of actions to mediate short-term motor learning. Cell *167*, 933–946.e20.
86. Lister, J.A., Robertson, C.P., Lepage, T., Johnson, S.L., and Raible, D.W. (1999). nacre encodes a zebrafish microphthalmia-related protein that regulates neural-crest-derived pigment cell fate. Development *126*, 3757–3767.
87. Macdonald, R. (1999). Zebrafish immunohistochemistry. In Molecular Methods in Developmental Biology: Xenopus and Zebrafish, M. Guille, ed. (Springer), pp. 77–88.
88. Lauter, G., Söll, I., and Hauptmann, G. (2011). Two-color fluorescent in situ hybridization in the embryonic zebrafish brain using differential detection systems. BMC Dev. Biol. *11*, 43.
89. Thisse, C., and Thisse, B. (2008). High-resolution in situ hybridization to whole-mount zebrafish embryos. Nat. Protoc. *3*, 59–69.
90. Sun, H., and Frost, B.J. (1998). Computation of different optical variables of looming objects in pigeon nucleus rotundus neurons. Nat. Neurosci. *1*, 296–303.

CellPress

STAR★METHODS

KEY RESOURCES TABLE

REAGENT or RESOURCE	SOURCE	IDENTIFIER
Antibodies		
p44/42 MAPK (Erk1/2) (L34F12) Mouse mAb	Cell Signaling Technology	Cat#4696; RRID: AB_390780
Rabbit anti-calretinin	Swant	Cat#7697; RRID: AB_2619710
Chemicals, Peptides, and Recombinant Proteins		
Dextran, Tetramethylrhodamine and biotin, 3000 MW, Lysine Fixable (micro-Ruby)	Thermo Fisher Scientific	Cat#D7162
CM-DiI Dye	Thermo Fisher Scientific	Cat#C7001
Critical Commercial Assays		
DIG RNA Labeling Kit	Sigma-Aldrich	Cat#11175025910
TSA Plus DNP (HRP) System	PerkinElmer	Cat#NEL747A001KT
TSA Plus Cyanine 3 System	PerkinElmer	Cat#NEL744001KT
TSA Fluorescein System	PerkinElmer	Cat#NEL701A001KT
TSA Cyanine 5 System	PerkinElmer	Cat#NEL705A001KT
NucleoSpin RNA Clean-up	MACHEREY-NAGEL	Cat#740948.50
Experimental Models: Organisms/Strains		
Tg(elavl3:H2B-GCaMP6s)jf5	[76]	ZFIN: ZDB-ALT-141023-2
ET(*gata2a*:EGFP)pku2Et	[77]	ZFIN: ZDB-ALT-080514-1
Tg(atoh7:GFP)rw021	[78]	ZFIN: ZDB-ALT-050627-2
Tg(Cau.Tuba1:c3paGFP)a7437Tg	[30]	ZFIN: ZDB-ALT-120919-1
Oligonucleotides		
chata riboprobe	[17]	N/A
chatb riboprobe	[17]	N/A
vachta riboprobe	[17]	N/A
vachtb riboprobe	[17]	N/A
hacta riboprobe	[17]	N/A
reelin riboprobe	[21]	N/A
gad1b riboprobe	[79]	N/A
vglut2b riboprobe	[80]	N/A
Software and Algorithms		
FIJI	N/A	http://fiji.sc
MATLAB	MathWorks	https://uk.mathworks.com/products/matlab.html
Advanced Normalization Tools (ANTs)	[81]	http://stnava.github.io/ANTs/
Zebrafish Brain Browser (ZBB)	[18]	https://science.nichd.nih.gov/confluence/display/burgess/Brain+Browser
NIfTI Input/Output (ImageJ plugin)	N/A	https://imagej.nih.gov/ij/plugins/download/jars/nifti_io.jar
Simple Neurite Tracer (ImageJ plugin)	[82]	https://imagej.net/Simple_Neurite_Tracer
LabVIEW	National Instruments	http://www.ni.com/en-gb/shop/labview.html
Psychophysics Toolbox	[83]	http://psychtoolbox.org/
Palamedes (MATLAB Toolbox)	[84]	http://www.palamedestoolbox.org/
MATLAB script for cell detection	[85]	https://github.com/ahrens-lab/Kawashima_et_al_Cell_2016/

CONTACT FOR REAGENT AND RESOURCE SHARING

Further information and requests for resources and reagents should be directed to and will be fulfilled by the Lead Contact, Isaac H. Bianco (i.bianco@ucl.ac.uk).

EXPERIMENTAL MODEL AND SUBJECT DETAILS

Animals were reared on a 14/10 h light/dark cycle at 28.5°C. For all experiments, we used zebrafish larvae homozygous for the *mitfa* skin-pigmentation mutation [86], except larvae used for single-cell electroporation, where 0.002% phenylthiourea (PTU) was added to the fish water from 12 hours-post-fertilization (hpf) to inhibit pigment formation. Larvae used for free-swimming behavior, retrograde DiI iontophoresis and calcium imaging experiments were Tg(*elavl3*:H2B-GCaMP6s)jf5 [76]. Single-cell electroporation experiments used double-transgenic larvae carrying Et(*gata2a*:EGFP)pku2 [77] and Tg(*atoh7*:GFP)rw021 [78] genomic features. Larvae used for behavior and functional imaging experiments were maintained in the Tuebingen background. All larvae were fed *Paramecia* from 4 dpf onward. The sex of the larvae is not defined at the early stages of development used for these studies. Experimental procedures were approved by the UCL Animal Welfare Ethical Review Body and the UK Home Office under the Animals (Scientific Procedures) Act 1986.

METHOD DETAILS

Whole-mount in situ hybridization and immunohistochemistry

Samples were fixed overnight in 4% paraformaldehyde (PFA) in 0.1 M phosphate buffered saline (PBS, Sigma-Aldrich) and 4% sucrose (Sigma-Aldrich) at 4°C. Brains were then manually dissected with forceps and fluorescent *in situ* hybridization (FISH) and immunostaining was performed as previously described [87, 88]. TSA Plus Cyanine 3 (Perkin Elmer, NEL744001KT) was used for single FISH and TSA Fluorescein System (Perkin Elmer, NEL701A001KT) and TSA Cyanine 5 System (Perkin Elmer, NEL705A001KT) were additionally used for triple FISH detection. mRNA anti-sense riboprobes for *chata, chatb, vachta, vachtb* and *hacta* were kindly provided by M. Halpern [17]. *reelin* [21], *vglut2b* [80] and *gad1b* [79] riboprobes were synthesized from plasmids [89]. For immunostaining, rabbit anti-Calretinin (Swant, Cat 7697, dilution 1:1000) and mouse anti-ERK (p44/42 MAPK (Erk1/2) 4696, 1:500) were used as primary antibodies followed by Alexa Fluor 488-conjugated (Molecular Probes, 1:200) secondary antibody.

Lipophilic dye tracing

Tg(*elavl3*:H2B-GCaMP6s) larvae were fixed as for the FISH protocol. Larvae were then mounted on a glass slide in drops of 2% low melting point agarose (Sigma) in PBS. Using a microsurgical blade, a small piece of agarose was cut away, exposing half of the head and eye. Micropipettes with a tip diameter of 1–2 μm were pulled on a P-87 micropipette puller (Sutter Instrument Company, CA) using AlSi glass capillaries containing a filament. Micropipettes were filled with a solution of the fluorescent carbocyanine dye CM-DiI (Thermo Fisher Scientific, C7001, 20 μg/mL in EtOH) and guided to either the OT or AF7 neuropil using a MX3000 Huxley-style micromanipulator (Soma Scientific Instruments) under water-immersion DIC optics (×40 objective, Axioskop 2 FS microscope, Carl Zeiss) and under fluorescent illumination (FITC filter). DC electrical stimulation was then briefly applied (1–5 s) using a 9 V alkaline battery and dye flow was directly observed under fluorescence. After dye application, specimens were unmounted and incubated in PBS in the dark at 4°C for a period of 7–10 days to allow the dye to diffuse. Imaging was performed using a laser scanning confocal microscopy (Leica TCS SPE or SP8, 488 nm and 461 nm excitation wavelength, HC FLUOTAR L 25x/0.95 W VISIR objective). Retrogradely labeled cells in the isthmus were counted manually using FIJI's Cell Counter plugin from the original image volumes. The *elavl3*:H2B-GCaMP6s imaging data was used to assess cell body staining and to register the images to the ZBB atlas. The coordinates of labeled cells were transformed to atlas reference space using the ANTs antsApplyTransformsToPoints function.

Focal electroporation and neuronal tracing

Focal electroporation was performed as described in [23]. Briefly, 4 dpf double transgenic Et(*gata2a*:EGFP);Tg(*atoh7*:GFP) larvae were anesthetized with tricane (0.02%, Sigma) and mounted on a custom-made slide. A micropipette was filled with a solution of fluorescent dextran (Dextran, Tetramethylrhodamine and biotin, 3000 MW, Lysine Fixable, Thermo Fisher Scientific, D7162, 0.2 mg/mL in dH_2O) and single cells were electroporated in the isthmus. After the procedure, larvae were unmounted and allowed to recover. At 6 dpf, larvae were anesthetized and imaged using laser scanning confocal microscopy (488 nm and 461 nm excitation). Single cell morphologies were traced using the Simple Neurite Tracer plugin for ImageJ [82].

3D image registration

Registration of image volumes was performed using the ANTs toolbox version 2.1.0 [81]. Images were converted to the NIfTi format, required by ANTs, using the ImageJ NIfTI Input/Output plugin. As an example, to register the sample image volume fish1–01.nii to the reference brain volume ref.nii, the following parameters were used:

antsRegistration -d 3 –float 1 -o [fish1_, fish1_Warped.nii.gz] –n WelchWindowedSinc -r [ref.nii, fish1–01.nii,1] -t Rigid[0.1] -m MI[ref.nii, fish1–01.nii,1,32, Regular,0.25] -c [200x200x200x0,1e-8,10] –f 12x8x4x2 –s 4x3x2x1-t Affine[0.1] -m MI[ref.nii,

fish1–01.nii,1,32, Regular,0.25] -c [200x200x200x0,1e-8,10] –f 12x8x4x2 –s 4x3x2x1-t SyN[0.1,6,0] -m CC[ref.nii, fish1–01.nii,1,2] -c [200x200x200x200x10,1e-7,10] –f 12x8x4x2x1 –s 4x3x2x1x0

The resultant deformation matrices were then applied to imaging channel N of fish1 using:

antsApplyTransforms -d 3 -v 0 –float -n WelchWindowedSinc -i fish1–0N.nii -r ref.nii -o fish1–0N_Warped.nii.gz -t fish1_1Warp.nii.gz -t fish1_0GenericAffine.mat

Registrations were typically performed using a Dell C6220 with 16 cores and 16 GB RAM. Registration was usually completed after 2–4 h for a template image with dimensions 1030 × 616 × 420 px, (pixel dimensions 1 × 1 × 1 μm x-y-z). All brains were registered onto the ZBB brain atlas [18], with some differences between experiments:

- For functional calcium imaging volumes a three-step registration was used: the imaging volume, which was usually composed of 2-7 image planes (500 × 500 px, 0.61 μm/px, 5 μm z-spacing), was first registered to a larger volume of the same brain taken after the experiment, using an affine transformation. Registration was manually inspected for each brain. Then, the larger volume was registered to a high-resolution whole-brain volume of a 6 dpf Tg(*elavl3*:H2B-GCaMP6s) transgenic, acquired using the same 2-photon microscope. This high-resolution volume was registered to the ZBB atlas. Consequently the transformations were concatenated to bring the functional imaging volume to the ZBB atlas (calcium volume → post-stack → hi-res → ZBB).
- For imaging volumes related to NI-ablations, the post-ablation volume was registered to the pre-ablation volume using an affine transformation. This step was done to ensure that the ablation ‘scar’, which was also apparent in the registration (green) channel, did not interfere with the following registration procedure. The pre-ablation volume was registered to the high-resolution *elavl3*:H2B-GCaMP6s brain. The post-ablation volume was then transported to the ZBB reference by concatenating the transformations (post-ablation → pre-ablation → hi-res → ZBB).
- For electroporation experiments, a reference volume was created by summing Et(*gata2a*:EGFP) and Tg(*atoh7*:GFP) volumes from the ZBB atlas. Imaging volumes from electroporated larvae were directly registered to this template.

All registrations were manually assessed for global and local alignment accuracy, particularly around the tectal neuropil, midbrain-hindbrain boundary and cerebellar tract adjacent to NI. To quantify registration accuracy in the isthmus, single cells were electroporated with fluorescent dextran conjugates in the right isthmus (n = 11 cells from 5 fish) of Et(*gata2a*:EGFP);Tg(*atoh7*:GFP) larvae and imaged as described above. Larvae were then unmounted from the agarose, remounted in a different position and imaged again. Both imaging volumes were then independently registered with identical parameters to the ZBB atlas. The distance between corresponding labeled cells in the resulting registered brains was computed to quantify registration error.

All brain regions referred to in this paper correspond to the volumetric binary image masks in the ZBB atlas, with the exception of newly defined regions in the isthmus (based on FISH and immunostaining), which were created by converting the median registered data for each molecular marker into a binary mask.

2-photon calcium imaging combined with behavioral tracking

The procedure was similar to that described in [13]. Tg(*elavl3*:H2B-GCaMP6s) larvae were mounted in agarose at 5 dpf and allowed to recover overnight before functional imaging at 6 dpf. Imaging was performed using a custom-built microscope [Olympus XLUMPLFLN ×20 1.0 NA objective, 580 nm PMT dichroic, bandpass filters: 510/84 (green), 641/75 (red) (Semrock), Coherent Chameleon II ultrafast laser]. Imaging was performed at 920 nm with average laser power at sample of 5–10 mW. Images (500 × 500 pixels, 0.61 μm/px) were acquired by frame scanning at 3.6 Hz and for each larva 2–5 focal planes were acquired with a z-spacing of 5 μm.

Stimuli were back-projected (Optoma ML750ST) onto a curved screen placed in front of the animal at a viewing distance of 35 mm while a second projector provided constant background illumination. Wratten filters (Kodak, no. 29) were placed in front of both projectors to prevent visual stimuli interfering with fluorescence detection. Visual stimuli were designed in MATLAB using Psychophysics toolbox [83]. For all experiments, stimuli were presented in a pseudo-random sequence with 30 s inter-stimulus interval. For the virtual hunting experiments, stimuli comprised 5˚ dark spots moving at 30˚/s either left–right or right–left across 152˚ of frontal visual space. Moving spots were presented in closed-loop such that if a convergent saccade was detected during stimulus presentation, spot position was updated to 0˚, simulating re-orientation toward the target [10]. Similar position updates were applied after each swim bout following a convergent saccade. In addition, 3 s whole-field light/dark flashes were presented. For looming experiments, expanding dark spots that simulated an object approaching at constant velocity were presented in front of the fish (10˚–70˚, L/V 490 ms) [90]. We also presented a control ‘dimming’ stimulus. This had a fixed angular size equal to the final size of the looming spot (70°) and dimmed so as to produce an identical change in whole-field luminance as the expanding looming spots.

Eye movements were tracked at 60 Hz under 720 nm illumination using a FL3-U3-13Y3M-C camera (Point Grey) that imaged through the microscope objective. Tail movements were imaged at 430 Hz under 850 nm illumination using a sub-stage GS3-U3-41C6NIR-C camera (Point Grey). Microscope control, stimulus presentation and behavior tracking were implemented using custom software written in LabView (National Instruments) and MATLAB (MathWorks).

Calcium imaging analysis

All data analysis was performed using custom-written MATLAB scripts. Motion correction of fluorescence imaging data was performed as per [13]. Regions of interest (ROIs) corresponding to cell nuclei were extracted using the cell detection code from [85] and fluorescence time-series for ROIs were computed by averaging pixel values within ROI binary masks for each frame. The time-varying fluorescence signal for each ROI was then standardized by 'z-scoring' and is denoted here as $F(t)$.

To compute visual response vectors, the mean F time-series was computed across the set of repetitions for each visual stimulus and these average responses were concatenated to produce the visual response vector (VRV) for the ROI. VRV clustering was performed similarly to [13] but using a multi-step procedure. In the first step, we performed hierarchical agglomerative clustering of VRVs using a correlation distance metric [13]. Clusters generated at a strict correlation threshold (0.9) were then used to define a set of archetypal cluster centroids. This was done by retaining only those members that fell within a threshold distance limit (0.5) of the centroid. Finally, VRVs that entered clusters in the first stage, but at a more lenient threshold (0.7), were assigned a cluster identity *de novo*. This was achieved by assigning the VRV to the closest archetypal centroid within a threshold distance limit (0.5).

Convergence Modulation Index (CMI) values were calculated for each cell as follows. For each GO trial, convergence-triggered activity was measured by computing the mean of $F(t)$ during a 4 s window centered on the convergent saccade, x_{GOi}. Next, activity was extracted at the same time and for the same visual stimulus, in NO-GO trials. The difference between GO and NO-GO activity was measured in units of the standard deviation of the NO-GO distribution:

$$d_i = \frac{X_{GOi} - \mu_{NO-GO}}{\sigma_{NO-GO}}$$

CMI values are computed as the mean of these d_i distance values across all GO trials during which the ROI was imaged. Sp-CMI values were computed by applying the same method for spontaneous convergences (those not during stimulus presentation). CMI and Sp-CMI were computed separately for 'left' convergent saccades (left eye displayed greater nasal rotation compared to right eye) and 'right' convergences (right eye shows larger nasal rotation), see [13].

To compare CMI values to those that might be expected by chance, a shuffle analysis was performed for each cell by circularly permuting its time-series $F(t)$ data (1000 times, each time by a random amount) and in each case recomputing CMI and Sp-CMI, as above.

Photoactivation of PA-GFP

Larvae (5 dpf) homozygous for Tg(*alpha tubulin*:C3PA-GFP) were anesthetized and mounted in 2% low-melting temperature agarose. A custom 2-photon microscope was used to photo-activate PA-GFP in a small region (9 × 9 μm) in the tectal *stratum periventricular* layer. The photo-activation site was selected by imaging the brain at 920 nm. Photo-activation was performed by continuously scanning at 790 nm (5 mW at sample), for 4 min. Larvae were then unmounted and allowed to recover. The following day, a post-conversion image stack (800 × 800 px, 0.385 μm/px, ~160 μm z-extent) was acquired at 920 nm covering a large proportion of the midbrain and tegmentum. Axonal projections were traced using the Simple Neurite Tracer plugin for ImageJ.

Laser ablations

Cell bodies in the isthmus of 6 dpf Tg(*elavl3*:H2B-GCaMP6s) larvae were targeted for ablation based upon their position with respect to the cerebellar tract that passes adjacent to NI. For sham ablations, a target region immediately medially adjacent to NI was targeted. Ablations were performed using the same custom-built 2-photon microscope described above. Briefly, a spiral scan was performed, centered on the target soma, for ~140 ms (800 nm, 150–200 mW at sample). An auto-fluorescent 'scar' was apparent on both green and red channels after successful ablation. Around 100 cells were targeted on each side of the brain and image stacks were acquired both before and after ablation. Fish were then unmounted and placed in single 35 mm Petri dishes with *Paramecia* to recover overnight. All fish survived the ablation procedure. Control fish were mounted alongside ablated fish and underwent the same manipulations except for laser-ablation.

Monitoring free-swimming behavior

The behavioral arena consisted of a 35 mm Petri dish which had been sanded on both internal and external walls to reduce reflections and covered on the outside with black tape. This was done to attempt to minimize thigmotaxis behavior. The dish was placed on a horizontal platform onto which visual stimuli could be projected (AAXA P2 Jr), via a cold mirror, from below. Images were acquired under 850 nm illumination using a high-speed camera (Mikrotron MC1362, 700 fps, 500 μs shutter-time) equipped with a machine vision lens (Fujinon HF35SA-1) and a 850 nm bandpass filter to block visible light. A solenoid 'tapper' was placed such that the piston, when extended, would contact the optomechanical frame of the rig. The solenoid was controlled using an Arduino Uno.

As before, visual stimuli were designed using Psychophysics Toolbox [83]. Looming stimuli expanded from 10–100° with L/V 255 ms. Varying contrast was achieved using equal increments of grayscale values against a constant background (464 lux): 100% (252 lux), 80% (268 lux), 60% (294 lux), 40% (334 lux), 20% (392 lux). Optomotor gratings had a period of ~10 mm and moved at 1 cycle/s. Because we tracked larvae online, optomotor gratings and looming spots could be presented in egocentric coordinates such that directional gratings always moved 90° to left or right sides with respect to fish orientation and looming spots were centered 5 mm away from the body centroid and at 90° to left or right. Stimuli were presented in pseudo-random order with an inter-stimulus

interval of minimum 90 s. Stimuli were only presented if the body centroid was within a predefined central region ('in middle', 11 mm from the edge of the arena). If this was not the case, a concentric grating was presented that drifted toward the center of the arena to attract the fish to the central region. After $\sim$1 h of testing, mechano-acoustic 'tap' stimuli were delivered with an inter-stimulus interval of 15 s for the final 10 min of the experiment.

At the beginning of each experiment, around 80 *Paramecia* were added to 3.5 mL of aquarium water in the arena. Initial *Paramecia* numbers were counted manually from full-frame video data from the first 5 min of the experiment. Larvae (7 dpf) were then added to the arena and allowed to acclimate for around 2 min before starting the experiment.

During experiments, eye and tail kinematics were tracked online as follows. First, images were background-subtracted using a continuously updated background model. Next, this image was thresholded and the 'body' centroid was found by running a particle detection routine for binary objects within suitable area limits. Eye centroids were detected using a second threshold and particle detection procedure with the requirement that these centroids were in close proximity to the body centroid. Body orientation and eye angles were computed from second and third central image moments. For both eyes, increases in eye angle indicate clockwise rotation. Vergence angle was computed as the difference between the left eye and the right eye angles. The tail was tracked by performing consecutive annular line-scans, starting from the body centroid and progressing toward the tip of the tail so as to define 9 equidistant x-y coordinates along the tail. Inter-segment angles were computed between the 8 resulting segments. Reported tail curvature was computed as the sum of these inter-segment angles. Rightward bending of the tail is represented by positive angles and leftward bending by negative angles. For the duration of the experiment, high-speed (700 Hz) video from a small window (length: 7.25 mm, 24.8 px/mm) centered on the fish was saved for offline scoring of hunting routines (see below). Additionally, full-frame video data was saved at 17.5 Hz for offline tracking of *Paramecia*. Camera control, online tracking and stimulus presentation were implemented using custom software written in LabView and MATLAB.

Free-swim behavior analysis

Data analysis was performed using custom MATLAB scripts. To identify periods of high ocular vergence, which represent hunting routines [9], a vergence angle threshold was computed for each fish by fitting two Gaussian models to its vergence angle distribution. This distribution is invariably bimodal and the vergence threshold was computed as one standard deviation below the center of the higher angle Gaussian. The fish was considered to be hunting if vergence angle exceeded this vergence threshold. Swim bouts were identified using a velocity threshold (500°/s) applied to smoothed cumulative tail angles. Escape responses to loom or tap stimuli were identified if instantaneous speed of the body centroid exceeded 75 mm/s. To track *Paramecia*, full-frame videos were smoothed using a 2D Gaussian filter (sigma = 7 px, 24.8 px/mm) and then segmented by finding local intensity maxima. Segmented particles were tracked using a frame-by-frame linking step using the Hungarian algorithm (simpletracker MATLAB script by Jean-Yves Tinevez, https://uk.mathworks.com/matlabcentral/fileexchange/34040-simple-tracker). To analyze contrast-dependence of loom-evoked escapes, logistic functions were fitted to escape probability data for each fish using the Palamedes MATLAB toolbox version 1.8.2 [84]. The lower asymptote was constrained to zero (spontaneous escapes were not observed). Goodness-of-Fit values were estimated by bootstrapping simulations (n = 1000).

Individual hunting epochs were manually examined and annotated using recorded high-speed videos. To identify target *Paramecia*, distance-gain and orientation-gain were computed for each swim bout in a hunting routine as the fraction of distance or orientation (azimuth) between fish and prey that was eliminated by that bout. Positive gain values indicate orienting toward and approaching the prey. For a *Paramecium* to be labeled as target, it was required to be located within the larva's reactive perceptive field at the beginning of the hunting routine ($\leq$6 mm from the center of the eyes and $\leq$ 120° from the frontal heading axis) and both distance-gain and orientation-gain for the first two bouts within the hunting sequence were positive with respect to that prey item. Fish-target distance was defined as the Euclidean distance between the center of the fish's eye centroids and the target centroid. Fish-target orientation was defined as the azimuth between the vector connecting the center of the fish's eyes to the target and the animals heading vector. Capture attempts were identified if the fish performed either a fast ram-like capture swim or a 'suction' [31]. Routines were labeled as aborted when no capture event occurred. A capture success was defined if the fish ingested the target. Target switches were identified if there was a clear re-orientation toward a different *Paramecium* during prey pursuit.

QUANTIFICATION AND STATISTICAL ANALYSIS

All statistical analyses were performed in MATLAB. Types of statistical test and *n* are reported in the text or figure legends. All tests were two-tailed and we report *p-value*s without correction for multiple comparisons, unless otherwise noted. Values in the main text are reported as mean ± SEM unless otherwise noted.

DATA AND SOFTWARE AVAILABILITY

Requests for datasets should be directed to and will be fulfilled by the Lead Contact, Isaac H. Bianco (i.bianco@ucl.ac.uk).

Current Biology, Volume *29*

Supplemental Information

Nucleus Isthmi Is Required to Sustain Target Pursuit during Visually Guided Prey-Catching

Pedro M. Henriques, Niloy Rahman, Samuel E. Jackson, and Isaac H. Bianco

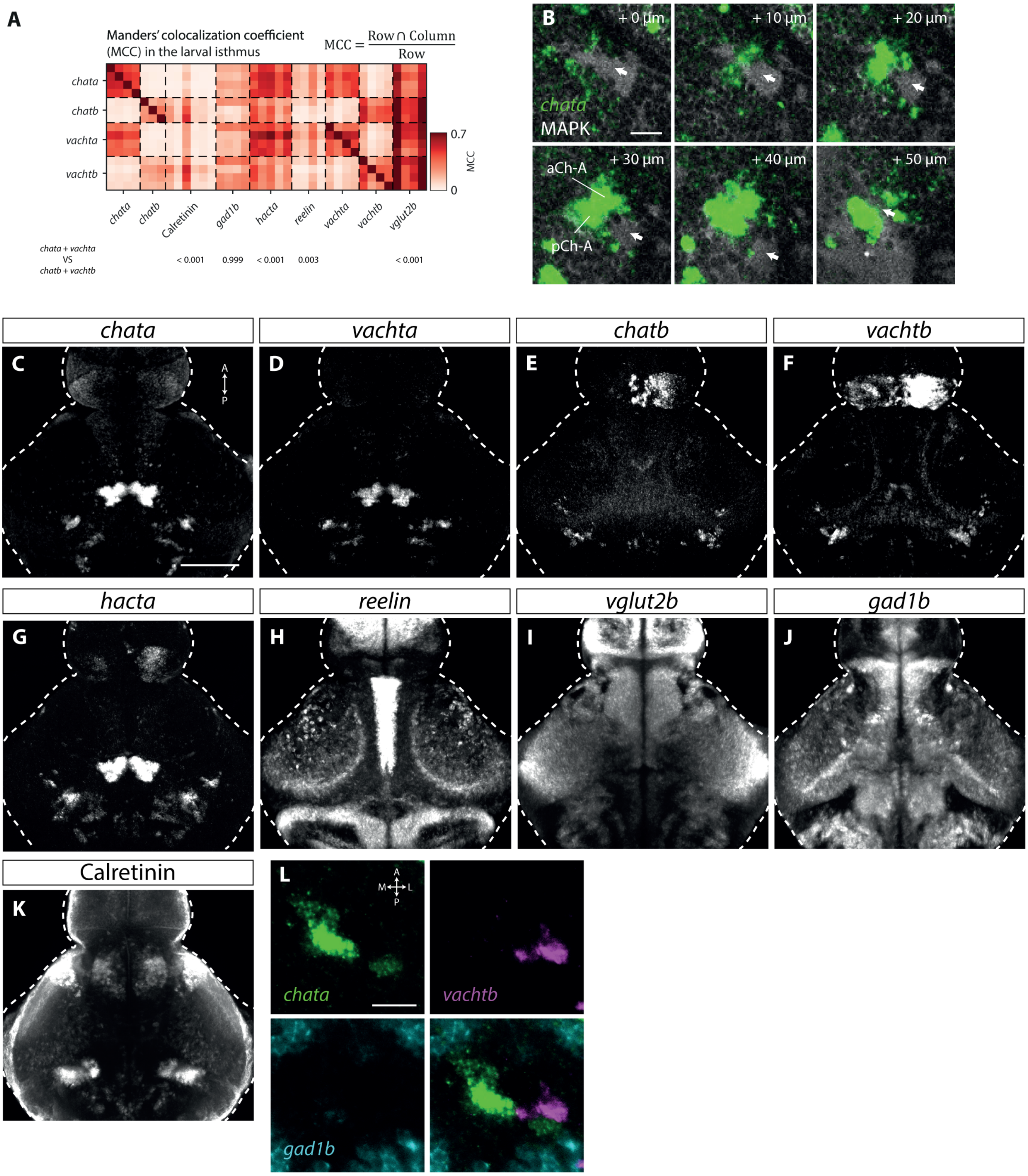

Figure S1: Isthmic gene expression, related to Figure 1. (A) Manders' colocalization coefficient (MCC) quantifying overlap of markers in the larval isthmus. This data shows all pairwise comparisons between single brains with mean values shown in Figure 1H. p-values correspond to comparisons of MCC for *chata+vachta* versus *chatb+vachtb* and the corresponding upper labels (Tukey's HSD correction for multiple comparisons following 2-way ANOVA). (B) MAPK expression in the isthmus showing the large cerebellar fascicle (white arrows) adjacent to and crossing *chata* expression. Scale bar, 25 μm. (C-K) Registered expression of markers used to characterize the larval isthmus. (C-H) show maximum-intensity projections across the entire dorso-ventral extent of the brain (325 μm), while (I-K) show projections across a smaller dorso-ventral region at the level of the isthmus (90 μm). All images show median intensity across registered datasets. Scale bar 100 μm. (L) Single focal plane from a triple fluorescent *in situ* of a 6 dpf larval brain detecting *chata*, *vachtb* and *gad1b* mRNA in the right isthmus. Almost no overlap is observed between these markers. Scale bar, 20 μm.

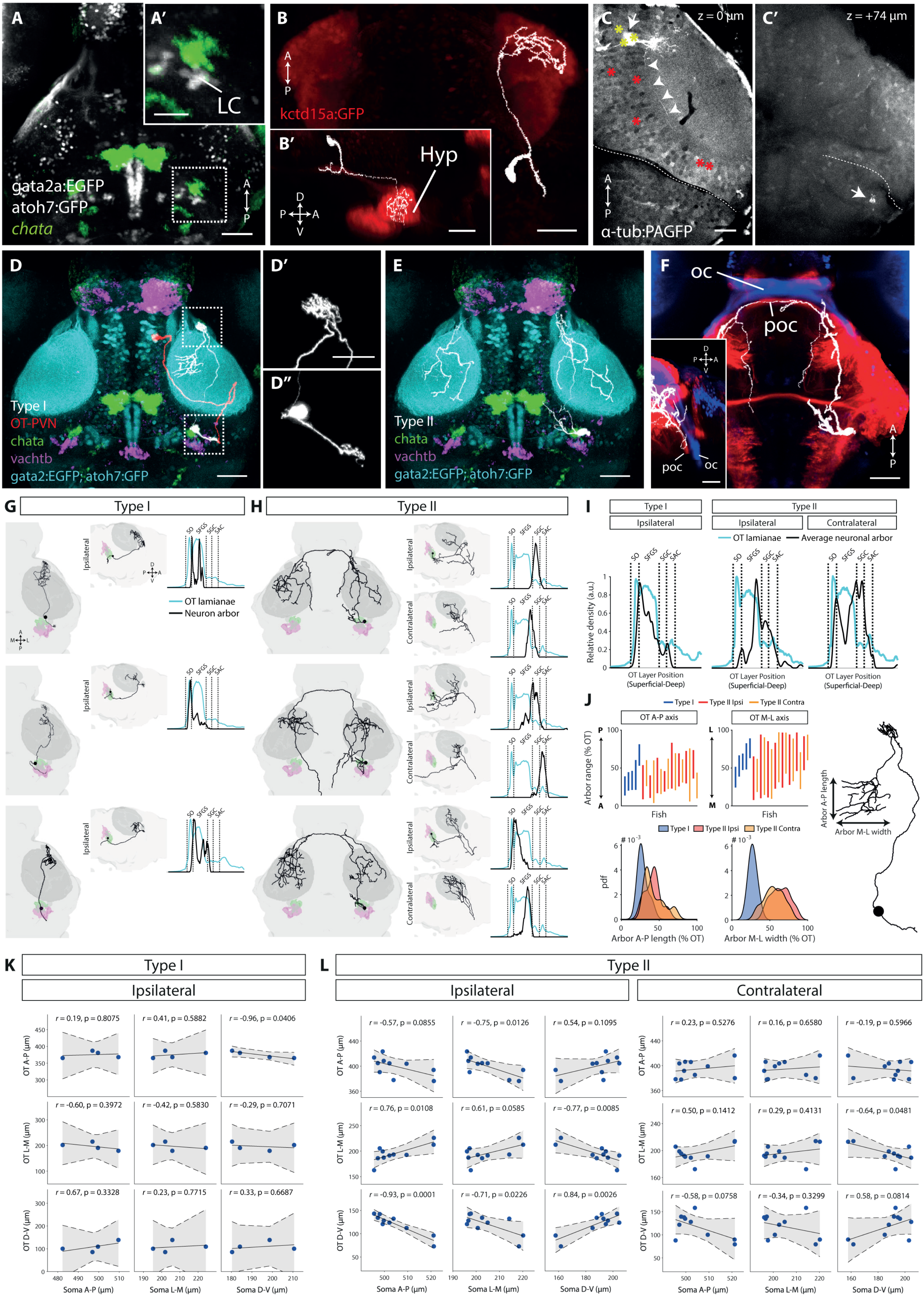
A
A'
LC
gata2a:EGFP
atoh7:GFP
chata
B
kctd15a:GFP
B'
Hyp
C
z = 0 µm
C'
z = +74 µm
α-tub:PAGFP
D
D'
D''
Type I
OT-PVN
chata
vachtb
gata2:EGFP; atoh7:GFP
E
Type II
chata
vachtb
gata2:EGFP; atoh7:GFP
F
OC
poc
oc
G
Type I
Ipsilateral
OT lamianae
Neuron arbor
SO
SFGS
SGC
SAC
H
Type II
Ipsilateral
Contralateral
I
Type I
Ipsilateral
Type II
Ipsilateral
Contralateral
OT lamianae
Average neuronal arbor
Relative density (a.u.)
OT Layer Position (Superficial-Deep)
J
Type I
Type II Ipsi
Type II Contra
OT A-P axis
OT M-L axis
Arbor range (% OT)
Fish
pdf
Arbor A-P length (% OT)
Arbor M-L width (% OT)
Arbor A-P length
Arbor M-L width
K
Type I
Ipsilateral
r = 0.19, p = 0.8075
r = 0.41, p = 0.5882
r = -0.96, p = 0.0406
r = -0.60, p = 0.3972
r = -0.42, p = 0.5830
r = -0.29, p = 0.7071
r = 0.67, p = 0.3328
r = 0.23, p = 0.7715
r = 0.33, p = 0.6687
OT A-P (µm)
OT L-M (µm)
OT D-V (µm)
Soma A-P (µm)
Soma L-M (µm)
Soma D-V (µm)
L
Type II
Ipsilateral
Contralateral
r = -0.57, p = 0.0855
r = -0.75, p = 0.0126
r = 0.54, p = 0.1095
r = 0.23, p = 0.5276
r = 0.16, p = 0.6580
r = -0.19, p = 0.5966
r = 0.76, p = 0.0108
r = 0.61, p = 0.0585
r = -0.77, p = 0.0085
r = 0.50, p = 0.1412
r = 0.29, p = 0.4131
r = -0.64, p = 0.0481
r = -0.93, p = 0.0001
r = -0.71, p = 0.0226
r = 0.84, p = 0.0026
r = -0.58, p = 0.0758
r = -0.34, p = 0.3299
r = 0.58, p = 0.0814
OT A-P (µm)
OT L-M (µm)
OT D-V (µm)
Soma A-P (µm)
Soma L-M (µm)
Soma D-V (µm)

Figure S2: NI Type I and Type II neurons, related to Figure 2. (A) Et(*gata2a*:EGFP);Tg(*atoh7*:GFP) double transgenic used for electroporation of cells in the larval isthmus. Maximum intensity projection overlaid with registered *chata* expression. Scale bar, 50 μm. (A') Isthmic region showing cells of the locus coeruleus (LC) in close proximity to *chata* expression. Scale bar, 25 μm. (B,B') Electroporated neuron in the isthmic region projecting to the hypothalamus, labelled by *kctd15a*:GFP [S1]. (B') shows lateral view. Scale bars, 50 μm. (C) Photo-activated PVNs in the rostral OT of a Tg(*alpha tubulin*:C3PA-GFP) larva. White arrow indicates location of photo-activation that resulted in labelling of several PVN somata. White arrowheads mark a single axon projecting from a photo-activated PVN. Yellow asterisks mark photo-activated regions in additional specimens that resulted in labelling of tectofugal projections to the ventrolateral anterior hindbrain, whereas red asterisks mark regions which did not. White dotted line indicates midbrain-hindbrain boundary. Scale bar, 25 μm. (C') Terminal arbor (white arrow) of the axon in (C), located in the ventrolateral anterior hindbrain. Complete traced projection is shown in panel (D), Figure 2A and Video S1. (D) Registered tracing of Type I neuron (white) and photo-activated OT PVN (red), overlaid with relevant markers. Same example cells as Figure 2A. Scale bar, 50 μm. (D',D") Magnified views of AF7 arborization and presumptive dendrite in hindbrain. (E) Registered tracing of Type II neuron (white), shown also in Figure 2B. (F) Type II axon decussating through the post-optic commissure (poc), marked by expression of *dbx1a*:Gal4 (red). The optic chiasm (oc), is located more rostrally and marked by *atoh7*:GFP (blue). Scale bar, 50 μm. Inset: Lateral view. (G-H) Registered tracings of Type I and Type II neurons, along with density profiles showing axon terminal distributions across tectal laminae. (I) Mean tectal lamination density profiles for all traced Type I ($n = 5$) and Type II ($n = 10$) cells. (J) Size and locations of Type I and Type II terminal arbors in OT. Bottom panel shows the distribution of arbor length and width as a fraction of tectal length/width. (K-L) Analysis of topographic innervation of OT. Locations of electroporated Type I ($n = 4$) and Type II ($n = 10$) cell bodies are plotted against centroid locations of terminal arbors in OT. We only considered datasets where there was no sign of registration error in any region of the brain and therefore one traced Type I cell was excluded due to registration uncertainty in the isthmic hindbrain. Linear fits to the data are shown with 95% confidence bounds. r, Pearson's correlation coefficient. Student's t-test.

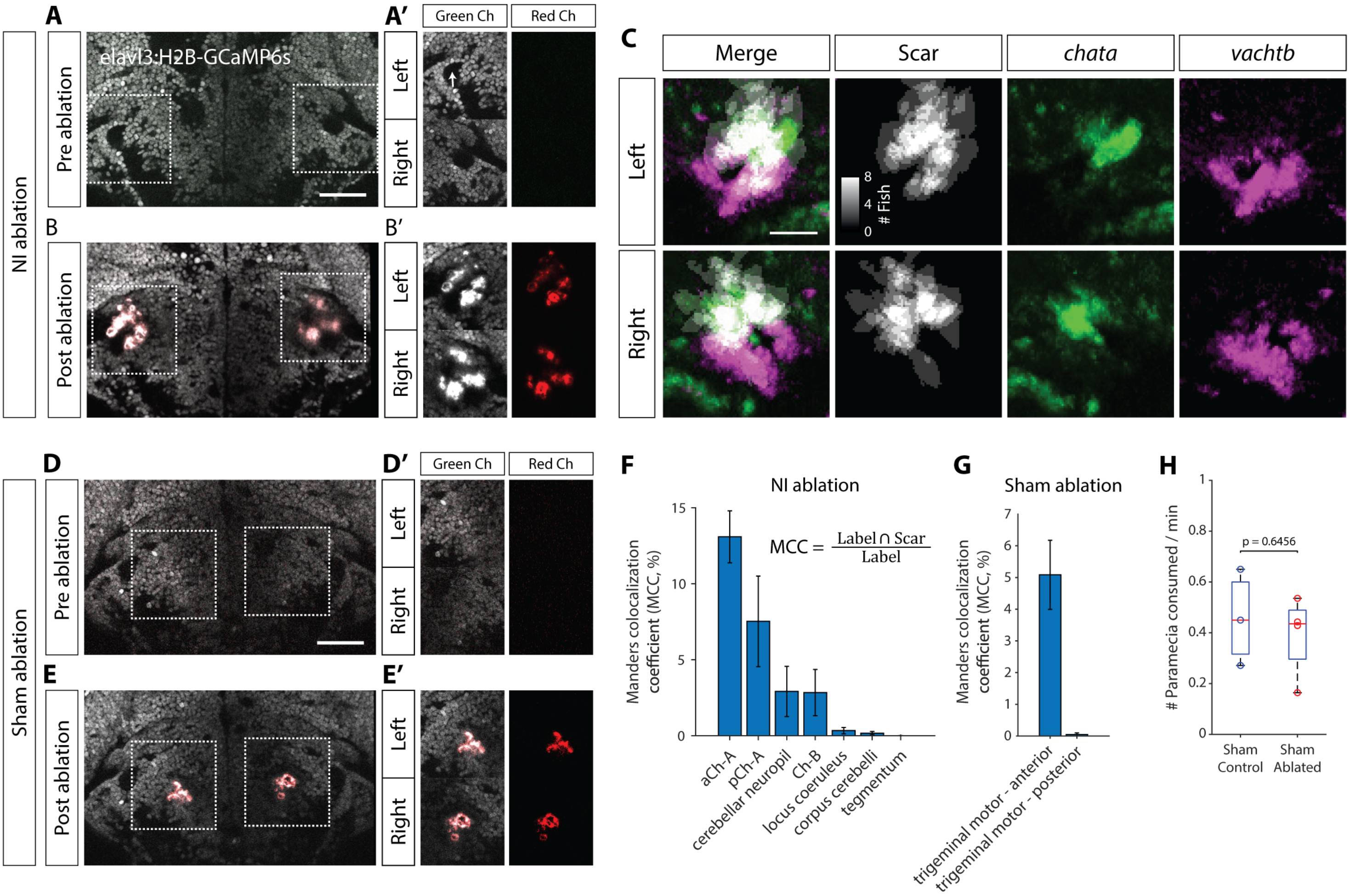

Figure S3: Laser ablations in isthmus, related to Figure 4. (A) 2-photon image of a 6 dpf Tg(*elavl3*:H2B-GCaMP6s) brain at the level of NI before ablation. Scale bar, 50 μm. (A') Isthmic region marked with dotted boxes in (A), showing green and red fluorescence channels. Before ablation there is no signal in the red channel. White arrow marks location of the cerebellar fascicle that can be used to locate NI. (B) Post-ablation image of the same brain. (B') The auto-fluorescent ablation scar can be seen in both green and red channels. (C) Sum of registered binary masks of ablation scars from 8 fish, overlaid with *chata* and *vachtb* expression. Scale bar, 25 μm. (D-E') Sham ablation of a region medially adjacent to NI. (F) Manders' colocalization coefficients (MCC) quantifying the percentage of voxels in ZBB brain regions that co-localize with the ablation scar for NI-ablations. Mean $\pm$ sem, $N = 8$ fish. (G) MCC quantification for sham ablation. (H) Prey consumption rates for sham-ablated larvae and their agarose-mounted sibling controls. Two sample t-test.

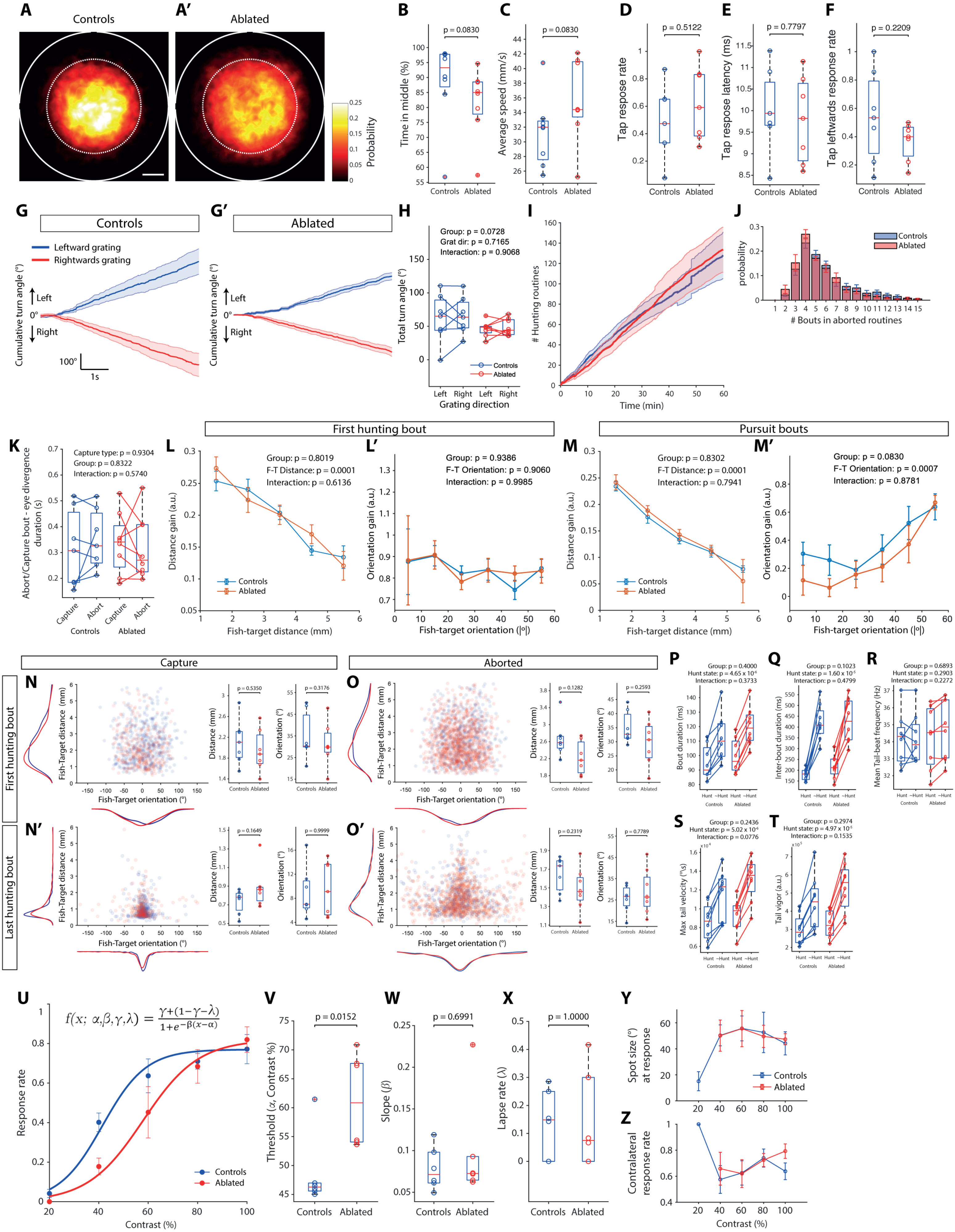
A
Controls
A'
Ablated
Probability
B
Time in middle (%)
p = 0.0830
C
Average speed (mm/s)
p = 0.0830
D
Tap respose rate
p = 0.5122
E
Tap response latency (ms)
p = 0.7797
F
Tap leftwards response rate
p = 0.2209
Controls Ablated
G
Controls
Leftward grating
Rightwards grating
Cumulative turn angle (°)
Left
Right
100°
1s
G'
Ablated
H
Group: p = 0.0728
Grat dir: p = 0.7165
Interaction: p = 0.9068
Total turn angle (°)
Grating direction
I
Hunting routines
Time (min)
J
probability
Bouts in aborted routines
K
Capture type: p = 0.9304
Group: p = 0.8322
Interaction: p = 0.5740
Abort/Capture bout - eye divergence duration (s)
Capture Abort Capture Abort
First hunting bout
L
Group: p = 0.8019
F-T Distance: p = 0.0001
Interaction: p = 0.6136
Distance gain (a.u.)
Fish-target distance (mm)
L'
Group: p = 0.9386
F-T Orientation: p = 0.9060
Interaction: p = 0.9985
Orientation gain (a.u.)
Fish-target orientation (|°|)
Pursuit bouts
M
Group: p = 0.8302
F-T Distance: p = 0.0001
Interaction: p = 0.7941
M'
Group: p = 0.0830
F-T Orientation: p = 0.0007
Interaction: p = 0.8781
Capture
Aborted
N
First hunting bout
Fish-Target distance (mm)
Fish-Target orientation (°)
p = 0.5350
p = 0.3176
Distance (mm)
Orientation (°)
O
p = 0.1282
p = 0.2593
N'
Last hunting bout
p = 0.1649
p = 0.9999
O'
p = 0.2319
p = 0.7789
P
Group: p = 0.4000
Hunt state: p = 4.65 x 10⁻⁶
Interaction: p = 0.3733
Bout duration (ms)
Q
Group: p = 0.1023
Hunt state: p = 1.60 x 10⁻⁵
Interaction: p = 0.4799
Inter-bout duration (ms)
R
Group: p = 0.6893
Hunt state: p = 0.2903
Interaction: p = 0.2272
Mean Tail-beat frequency (Hz)
S
Group: p = 0.2436
Hunt state: p = 5.02 x 10⁻⁶
Interaction: p = 0.0776
Max tail velocity (°/s)
T
Group: p = 0.2974
Hunt state: p = 4.97 x 10⁻⁵
Interaction: p = 0.1535
Tail vigor (a.u.)
Hunt ~Hunt Hunt ~Hunt
U
f(x; α,β,γ,λ) = (γ+(1−γ−λ))/(1+e^{−β(x−α)})
Response rate
Contrast (%)
V
p = 0.0152
Threshold (α, Contrast %)
W
p = 0.6991
Slope (β)
X
p = 1.0000
Lapse rate (λ)
Y
Spot size (°) at response
Z
Contralateral response rate
Contrast (%)

Figure S4: Behavior of NI-ablated larvae, related to Figure 4. (A,A') Distribution of control and NI-ablated larvae in the behavioral arena. Outer white circle marks edge of the arena and inner dotted circle shows the area in which larvae were considered 'in middle' for triggered presentation of visual stimuli. For each fish, a 2D probability histogram was generated for the duration of the experiment and convolved with a 2D gaussian filter (sigma = 15 px, 24.8 px/mm). Map shows mean probability density across fish. Scale bar, 5 mm. (B) Percentage of time larvae spent 'in middle' of the arena. (C) Average speed of the larvae over the duration of the experiment. (D-F) Response rate, latency and leftward response probability for mechano-acoustic 'tap'-evoked escapes. Data points are median values for each fish. Wilcoxon rank sum tests. (G-G') Cumulative turn angle in response to directional drifting gratings (mean $\pm$ sem). (H) Total cumulative turn angle during period of the grating stimulus. Two-way repeated measures ANOVA. (I) Cumulative number of hunting routines initiated by NI-ablated and control larvae over the course of the experiment. (J) Distribution of number of bouts within aborted hunting routines. (K) Time interval from end of capture swim or negative-gain (abort) bout to eye divergence. Data points are median per fish. Two-way repeated measures ANOVA. (L-M') Distance- and orientation-gain for first bout in hunting sequence and subsequent prey-tracking bouts. Data was binned based on prey orientation/distance. Mean $\pm$ sem of median values for each fish. Two-way ANOVA. (N-O') Spatial locations of target *Paramecia* at hunting initiation and immediately prior to capture swims (N') or at termination of aborted routines (O'). Data points and marginal histograms show data for control (blue) and ablated (red) larvae. Wilcoxon rank sum tests. (P-T) Motor kinematics for swim bouts within hunting routines ('Hunt') or when fish were not hunting ('$\sim$Hunt'). Data points represent median values for each fish. Two-way repeated measures ANOVA. (U) Response rates to looming stimuli of varying contrast. Curves show psychometric fits for control ($N = 6$) and NI-ablated ($N = 6$) groups. Note that 5 control and 4 ablated larvae were also tested for performance in hunting and other behaviors (Figure 4 and panels A-T) whereas the remaining larvae were tested only for loom-avoidance. (V-X) Quantification of psychometric fit parameters for individual fish. Wilcoxon rank sum tests. (Y) Angular size of looming spot at time of escape response. (Z) Frequency of escapes directed away from the location of the looming stimulus. Lack of data at 20% contrast in the NI-ablated group is due to the fact that no fish responded at this contrast level. Data presented as mean $\pm$ sem.

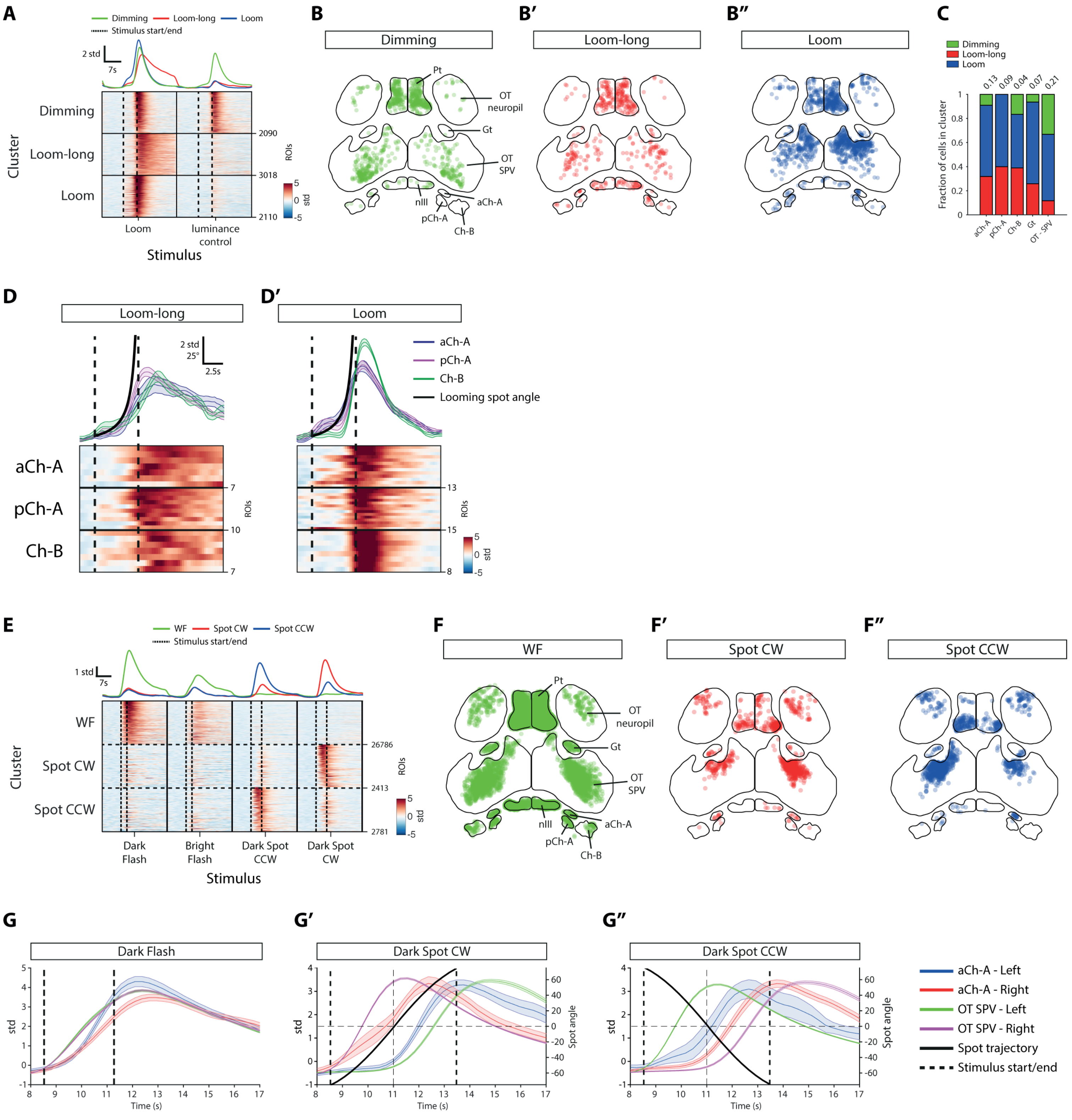

Figure S5: Visually evoked neural activity, related to Figure 5. (A) Clustered VRVs for the looming experiment. Top shows average responses of each cluster. Rows in the raster show VRVs for all clustered cells. The number of neurons in each cluster is shown on the right. (B-B'') Anatomical distribution of cells assigned to visual clusters in the looming experiment. (C) Visual cluster assignments for each brain area. Numbers on top show the fraction of cells in the brain region that were assigned cluster identities. (D,D') Responses of NI neurons in the Loom and Loom-long clusters. (E) All clustered VRVs for hunting experiment. (F-F'') Locations of visually responsive cells in the hunting experiment. (G-G'') Fluorescent time-series of aCh-A and OT-SPV neurons to prey-like visual stimuli (mean $\pm$ sem). Dashed lines indicate period of stimulus presentation and horizontal and vertical dotted lines indicate when the moving spot is at the centre of the screen (directly ahead of the fish). Negative spot angles correspond to the left visual hemifield. nIII, oculomotor nuclei; OT SPV, optic tectum stratum periventriculare; Gt, griseum tectale; Pt, pretectum.

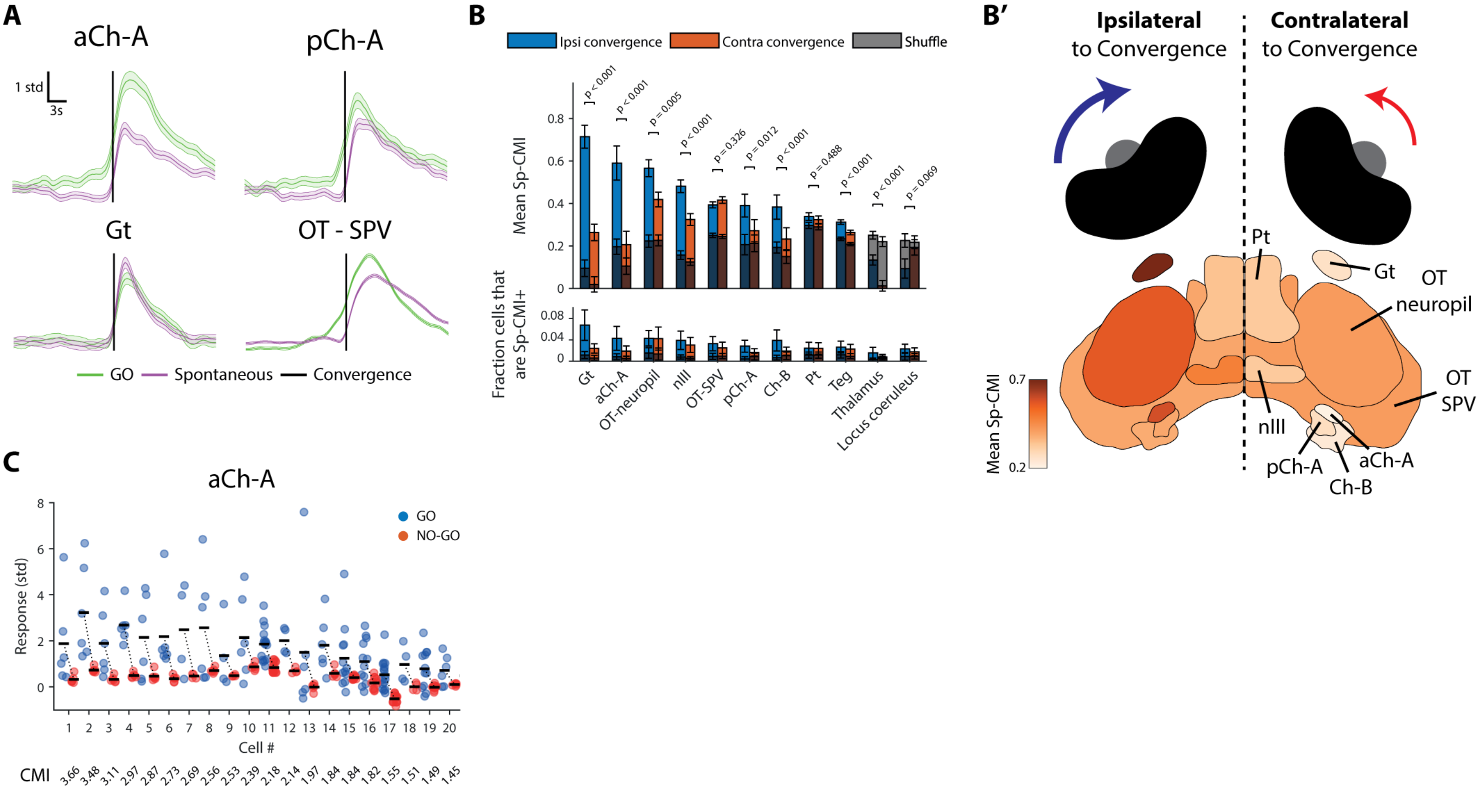

Figure S6: Convergence-related neural activity, related to Figure 6. (A) Average normalized fluorescence response of aCh-A, pCh-A, Gt and OT-SPV neurons triggered on time of convergent saccades (mean ± sem). (B,B') Sp-CMI scores. Mean Sp-CMI and fraction of Sp-CMI+ cells for several imaged brain regions. Mean ± sem. Wilcoxon signed rank tests between each region's ipsilateral and contralateral convergence distributions. (B') shows brain areas colored by mean Sp-CMI scores for ipsilateral and contralateral convergences. (C) Responses of individual aCh-A cells across the complete set of GO and NO-GO trials during which their calcium activity was recorded. Twenty cells with varying CMI scores are displayed, sorted by descending CMI.

Supplemental Reference

S1. Heffer, A., Marquart, G.D., Aquilina-Beck, A., Saleem, N., Burgess, H.A., and Dawid, I.B. (2017). Generation and characterization of Kctd15 mutations in zebrafish. PloS One 12, e0189162.
